# Lipopolysaccharide O-antigen delays plant innate immune recognition of *Xylella fastidiosa*

**DOI:** 10.1038/s41467-018-02861-5

**Published:** 2018-01-26

**Authors:** Jeannette N. Rapicavoli, Barbara Blanco-Ulate, Artur Muszyński, Rosa Figueroa-Balderas, Abraham Morales-Cruz, Parastoo Azadi, Justyna M. Dobruchowska, Claudia Castro, Dario Cantu, M. Caroline Roper

**Affiliations:** 1 Department of Microbiology and Plant Pathology, University of California, Riverside, CA 92521 USA; 2 Department of Viticulture and Enology, University of California, Davis, CA 95616 USA; 3 Department of Plant Sciences, University of California, Davis, CA 95616 USA; 4 Complex Carbohydrate Research Center, University of Georgia, Athens, GA 30602 USA

## Abstract

Lipopolysaccharides (LPS) are among the known pathogen-associated molecular patterns (PAMPs). LPSs are potent elicitors of PAMP-triggered immunity (PTI), and bacteria have evolved intricate mechanisms to dampen PTI. Here we demonstrate that *Xylella fastidiosa* (*Xf*), a hemibiotrophic plant pathogenic bacterium, possesses a long chain O-antigen that enables it to delay initial plant recognition, thereby allowing it to effectively skirt initial elicitation of innate immunity and establish itself in the host. Lack of the O-antigen modifies plant perception of *Xf* and enables elicitation of hallmarks of PTI, such as ROS production specifically in the plant xylem tissue compartment, a tissue not traditionally considered a spatial location of PTI. To explore translational applications of our findings, we demonstrate that pre-treatment of plants with *Xf* LPS primes grapevine defenses to confer tolerance to *Xf* challenge.

## Abstract (web summary)

Many pathogenic bacteria have evolved to subvert host immune responses triggered by lipopolysaccharides (LPS). Here the authors show that a long terminal polysaccharide chain, known as the O-antigen, present in LPS from the plant pathogen *Xylella fastidiosa* can delay recognition by grapevine hosts.

## Introduction

Unlike mammals, plants lack mobile defender cells and an adaptive somatic immune system. Alternatively, they rely on innate immunity and signal transduction pathways emanating from infection sites^1^. The first tier of the immune system functions through recognition of pathogen- (or microbe-) associated molecular patterns (PAMPs/MAMPs) resulting in PAMP-triggered immunity (PTI)^1^. There are numerous pathogenesis-related changes that follow PAMP perception, such as rapid influxes of cytosolic Ca^+2^ and production/accumulation of reactive oxygen species (ROS). Additional downstream responses that occur within hours of PTI initiation are stomatal closure and cell wall modifications such as callose deposition^2^. Studies on the molecular mechanisms underlying PTI have primarily focused on foliar pathogens that enter through epiphytic means^3^. There is little information on the initiation of PTI by microbes that reside in the vascular tissue, particularly in the xylem, where microorganisms come into contact with mostly non-living tissue.

Many bacterial vascular plant pathogens enter host tissues passively (via wounds, cracks, or natural openings)^4,5^, but some are exclusively delivered directly into the plant vascular system by insect vectors, which is the case for the bacterium *Xylella fastidiosa* (*Xf*), the causal agent of Pierce’s disease (PD) of grapevine^6^. Following infection with xylem-dwelling pathogens, plant hosts deploy defense responses that include both physical (e.g., tyloses) and metabolic compounds/proteins related to defense (e.g., phenolics, PR proteins, phytoalexins, and peroxidases) that aim to halt pathogen spread or inhibit/kill pathogen growth. Xylem cells undergo programmed cell death and are non-living at maturity and, thus, do not mount these defense responses on their own^4,5,7,8^. Vascular pathogens are presumably recognized by receptors in the living parenchyma cells surrounding the xylem^4,7^. Perception of the xylem-dwelling bacterial pathogens via receptor-mediated detection of secreted type III effectors is well-documented^7^, but the mechanisms of PAMP perception in the xylem, particularly in regards to PTI, are unclear.

*Xf* is a Gram-negative, xylem-limited bacterial phytopathogen that is obligately transmitted by leafhopper vectors, mainly sharpshooters^9^. *Xf* exclusively occupies the xylem, making it a robust model system for understanding xylem-specific plant–microbe interactions^10^. This destructive pathogen causes diseases in several economically important crops, namely PD of grapevine, citrus variegated chlorosis, and almond leaf scorch, and it has recently been implicated in olive quick decline syndrome^10,11^. The bacteria colonize the xylem vessels of plant hosts and elicit production of prolific host-derived xylem occlusions (i.e., tyloses) that reduce hydraulic conductivity in the plant^12,13^. Studies centered on the late stages of infection indicate transcriptional changes occurring that include responses to abiotic drought stress^14^. However, details regarding the elicitation of basal defense responses in the very early phases of host infection remain largely unknown, as well as the dynamics of the plant’s response to *Xf* over time.

Lipopolysaccharides (LPSs) are abundant structural components of the cell envelope of most Gram-negative bacteria^15^. LPSs are comprised of three parts: (i) lipid A that anchors the molecule to the outer membrane, (ii) a core oligosaccharide (OS), and (iii) a terminal O-antigen polysaccharide chain^15^. The O-antigen is the most surface-exposed portion of the LPS molecule and can extend up to 30 nm away from the surface of the bacterial cell^16–18^. Although the lipid A and OS portions of the molecule are highly conserved, the O-antigen can exhibit considerable structural variation^17,19,20^. The O-antigen is assembled in the cytoplasm and delivered to the periplasm, where it is ligated onto the lipid A–OS complex and then translocated to the outer membrane^17^. This is mediated, in part, by the Wzy polymerase, which catalyzes the polymerization of the individual O-units that make up the O-antigen chain^17^. *Xf* possesses a high molecular weight rhamnose-rich O-antigen, and a deletion mutation in *wzy* results in a severely truncated O-antigen. This truncation of the O-antigen compromised *Xf’s* ability to colonize the grapevine xylem and elicit PD symptoms over the course of the long infection process (18 weeks)^21^.

Purified LPSs from diverse bacterial pathogens induce a rapid oxidative burst (a hallmark of the elicitation of PTI) in *Arabidopsis thaliana*^22^, and LPS from various plant and animal pathogens can induce defense responses in rice cells^23^. LPS can also induce PR gene expression and callose deposition^24,25^. Many plant and animal bacterial pathogens dampen PTI by secreting type III effectors that disrupt or suppress PTI responses^26,27^. Interestingly, a type III secretion system is absent in *Xf*^28^, and there is no known mechanism of suppressing PTI for this bacterium. However, the bacterial cells somehow overcome PTI to successfully establish themselves in the host, as *Xf* moves systemically in grapevines and achieves very high titers over the course of the long growing season in this perennial host. This mechanism(s) of overcoming PTI is unknown but could range from secretion of PTI-dampening effectors from other secretion systems (such as type I, II IV, or V) or by physically masking the infectious cells with a cell surface entity that, instead of dampening PTI, would allow the cells to delay elicitation of PTI. Here, we establish that *Xf* synthesizes a high molecular weight O-antigen that effectively masks the cells to delay PTI in the grapevine host. Infection processes are very dynamic, and there are many facets to the bacteria–plant immune system interface. This shielding strategy is one mechanism that contributes to *Xf* establishment within the xylem and, ultimately, pathogenesis.

## Results

### Extracted LPSs induce an oxidative burst from leaf discs

**Fig. 1:**
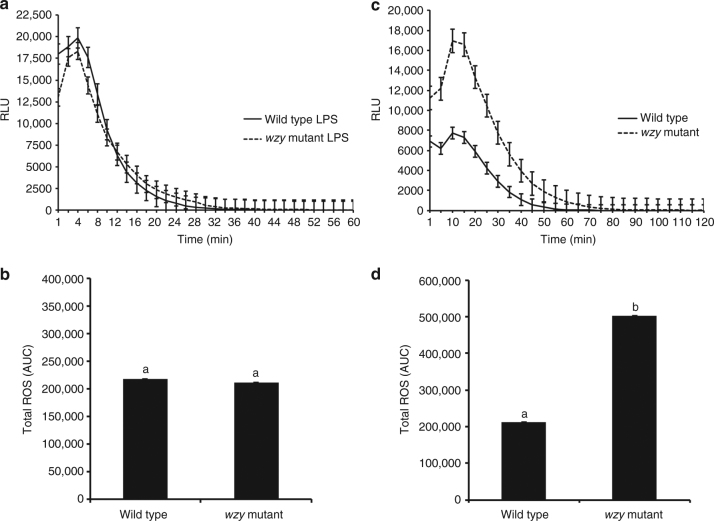
O-antigen-modulated ROS production by extracted LPS and intact bacterial cells ex vivo. Discs of *V. vinifera* ‘Cabernet Sauvignon’ leaves were treated with 20 μL of a 50 μg/mL solution of purified LPS elicitors (either *wzy* or wild type LPS) equal to a final amount of 10 μg (based on Kdo content) of LPS, 20 μL of a 10^8^ CFU/mL suspension of *Xf* wild type or *wzy* cells, or diH_2_0 or 1× PBS-inoculated controls, respectively. **a** The amplitude of ROS production remained similar for both wild type and *wzy* LPS, reaching max production at ~4 min, and plateaued starting around 30 min. **b** Total ROS production is reported as area under the curve (AUC) for plot of luminescence intensity over time. Total ROS production was not significantly different between discs treated with wild type or *wzy* extracted LPS. **c** Intact *wzy* cells induced a significantly stronger oxidative burst that persisted nearly 20 min longer than leaves inoculated with wild type bacteria (which contained fully polymerized O-antigens). Graphs represent the mean of 24 replicates per treatment ± standard error of the mean. **d** Total ROS production is reported as area under the curve (AUC) for plot of luminescence intensity over time. Discs treated with *wzy* cells produced significantly more ROS than discs treated with wild type cells. Graphs represent the mean of 24 replicates per treatment ± standard error of the mean. Treatments with different letters over the bars are statistically different (*P* < 0.05, Mann–Whitney *U* test, *n* = 24 per treatment)

Using a ROS burst as the indicator, we initially sought to assess if the *Xf* LPS functions as a PAMP in grapevine. We tested LPS extracted from *Xf* cells to determine LPS PAMP activity independent of other interacting components inherent to biological cells. Using an ex vivo luminol-based assay to demonstrate the kinetics of the ROS burst, LPSs extracted from both wild type and *wzy* cells (10 μg final amount, based on Kdo content) induced an oxidative burst of similar amplitude from grapevine leaf discs (Fig. 1a), and total ROS production was not significantly different between the treatments (*P* < 0.05, one-way ANOVA, Mann–Whitney *U* test, *n* = 24 per treatment) (Fig. 1b).

We also demonstrate that LPS preparations purified in the same manner as the O-antigen structural studies of *Xf* LPS (nuclease, protease treatment, dialysis and ultracentrifugation) elicit ROS using an Amplex Red hydrogen peroxide detection assay. Indeed, both wild type and *wzy* LPSs in these purified forms elicit ROS production from grapevine leaf discs and there was no statistical difference (*P* < 0.05, two-way ANOVA, Tukey’s adjustment, *n* = 5 per treatment) in ROS production between LPS type (wild type or *wzy*) (Supplementary Figure 1), similar to what we observed for extracted LPSs (Fig. 1a, b). The effect of concentration between treatments was also not significant (*P* < 0.05, two-way ANOVA, Tukey’s adjustment, *n* = 5 per treatment), indicating that ROS production did not differ between wild type and *wzy* treatments regardless of LPS concentration. Interestingly, the amount of ROS production did not increase linearly when LPS dose amount increased. The multiple comparisons show that ROS production is not significant among 5 µg, 10 µg, and 12.5 µg LPS doses. The only significant difference was between 10 µg and 20 µg with 20 µg of LPS eliciting significantly less ROS production than 10 µg (*P* < 0.05, two-way ANOVA, Tukey’s adjustment, *n* = 5 per treatment). We speculate that this could be due to micelle formation/aggregation of LPS at higher concentrations or saturation of the unknown grapevine LPS receptor when 20 µg of LPS is applied to the disc.

### Intact *wzy* cells elicit a more intense oxidative burst

In intact *Xf* cells, much of the lipid A–core complex would be partially embedded in the outer membrane. Thus, to explore the effects of O-antigen structure on the elicitation of PTI in the biological context of whole cells, we evaluated *Xf* cell-induced production of ROS ex vivo. Although both wild type and *wzy* cells induced oxidative bursts from the leaf discs, the mutant triggered a more intense oxidative burst, with ROS production peaking around 10 min and persisting nearly 80 min (Fig. 1c). The wild type bacteria failed to produce a sharp peak of ROS as compared to the *wzy* bacteria, and production plateaued around 60 min. In addition, total ROS production was significantly larger in response to *wzy* cells as compared with wild type (*P* < 0.05, one-way ANOVA, Mann–Whitney *U* test, *n* = 24 per treatment) (Fig. 1d).

### O-antigen-modulated ROS occurs in xylem vessels

**Fig. 2:**
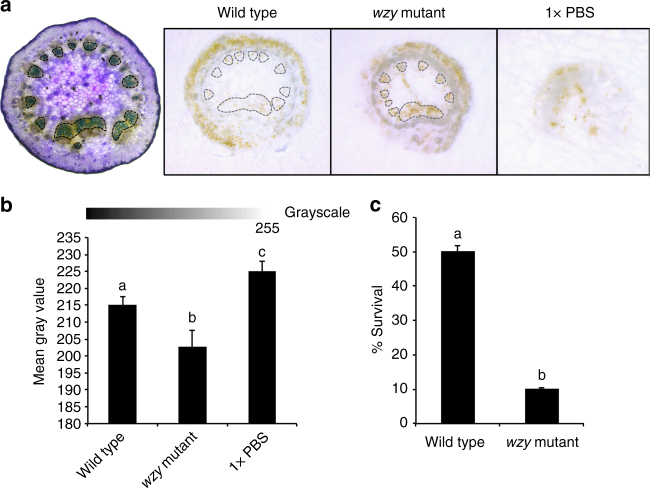
In situ localization of O-antigen-modulated ROS production in the xylem in petioles of inoculated plants. **a** DAB-mediated tissue printing of petioles at 15 min post-inoculation indicated a strong production of H_2_O_2_ specifically in the xylem vessels of grapevines needle inoculated with *wzy* cells (location of xylem vessels emphasized with dotted outline). Vines inoculated with wild type *Xf* exhibited H_2_O_2_ production predominantly in peripheral collenchyma tissue, with some production in the xylem vessels. Vines inoculated with 1× PBS buffer served as negative controls. **b** Mean gray value of grayscale-converted DAB-stained images, representing differences in staining intensity. Grayscale intensities vary from 0 to 255; 0 = black, 255 = white, with the values in between representing shades of gray. The mean gray value of DAB-stained images from *wzy*-inoculated plants is significantly lower than wild type or 1× PBS-inoculated plants, indicating a darker, or more intense stain, and thus higher amounts of H_2_O_2_. Treatments with different letters over the bars are statistically different (*P* < 0.05, one-way ANOVA, Tukey’s adjustment, *n* = 9 per treatment). Error bars represent ± standard error of the mean. H_2_O_2_ survival assay. **c** Suspensions of *Xf* wild type or *wzy* cells were incubated with a final concentration of 100 μM H_2_O_2_ for 10 min, followed by dilution plating and enumeration. Survival percentages of *wzy* cells were significantly lower than *Xf* wild type cells (*P* < 0.0001, Mann–Whitney *U*, *n* = 9 per treatment). Following treatment with H_2_O_2_, 10.1 and 50.2% of *wzy* and wild type cells survived, respectively. Data are means of three biological replications and error bars represent ± standard error of the mean

To determine where ROS production was localized when *Xf* was inoculated into whole grape plants (rather than applied to leaf discs ex vivo), we performed an additional in vivo experiment where we inoculated grapevines with wild type *Xf*, *wzy* cells, or 1× PBS buffer. At 15 min post-inoculation, we harvested the petiole closest to the point of inoculation and immediately performed DAB (3,3*′*-diaminobenzidine)-mediated tissue printing of these excised petioles. DAB precipitation is indicative of the spatial location of ROS in the tissue. In comparison to grapevines inoculated with wild type cells, grapevines inoculated with *wzy* cells exhibited more intense H_2_O_2_ production prominently localized in the xylem vessels (Fig. 2a, dotted outline). Quantitative comparison of staining intensity among the treatments using ImageJ indicated that, indeed, *wzy* elicits a more intense production of ROS in the xylem than does wild type *Xf* (*P* < 0.05, one-way ANOVA, Tukey’s adjustment, *n* = 9 per treatment) (Fig. 2b).

### H_2_O_2_ survival test

The *wzy* mutant is more sensitive to H_2_O_2_^21^, and here we show the approximate concentration of H_2_O_2_ that bacterial cells are exposed to in the xylem affects survivability. We chose a final concentration of 100 μM H_2_O_2_ based on the lower threshold of ROS detected by the DAB staining method (DAB staining detects H_2_O_2_ in the range of 100 μM–10 mM)^29^. To mirror the kinetics of peak ROS production seen in vivo, we exposed the cells to H_2_O_2_ for 10 min. Both wild type and *wzy* cells were affected by treatment with H_2_O_2_, but significantly fewer *wzy* cells survived (10.1%), compared to wild type cells (50.2%) (Fig. 2c).

### Transcriptomic analyses of early responses to *Xf*

**Fig. 3:**
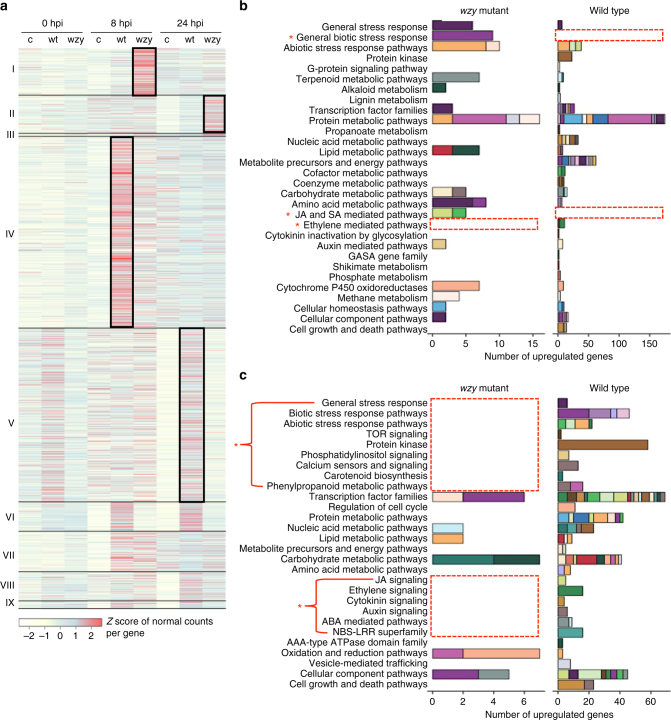
Grapevine responses to early infection by *wzy* mutant or wild type *Xf*. **a** Upregulated grape genes (*P* < 0.05) in response to *wzy* mutant (wzy) or wild type (wt) bacteria at 8 and 24 h post-inoculation (hpi) when compared to 1× PBS controls (c). Genes are classified into nine groups (I–IX) based on their expression pattern. The colors in the heat map represent the *Z* score of the normal counts per gene, and black boxes represent gene groups distinctly upregulated in response to each treatment and time point. **b** Enriched grape functional pathways (*P* < 0.05, hypergeometric test, *n* = 3 per treatment) among genes upregulated during *wzy* (group I) or wt (group IV) infections at 8 h. **c** Enriched grape functional subcategories (*P* < 0.05, hypergeometric test, *n* = 3 per treatment) among genes upregulated during *wzy* (group II) or wt (group V) infections at 24 h. Colored stacked bars represent individual pathways. Red boxes highlight functions of interest (*) that are enriched in one treatment, but not enriched in the other at each time point. The complete dataset, including the color legend for each pathway, is available in Supplementary Data 3

To elucidate the early plant immune responses to whole *Xf* cells on the global transcriptional level, we performed a series of RNAseq experiments on grapevines inoculated with wild type, *wzy* cells, or 1× PBS buffer (controls). In previous studies, transcript accumulation of specific laminarin-induced defense genes in grapevine were not detectable until 10 h post-treatment, with some genes not reaching peak expression until 20 h post-treatment^30^. Thus, in an effort to encompass a range of potential grapevine defense responses, RNAseq analyses were conducted using petioles collected at 8 and 24 h post-inoculation. A summary of the parsing and mapping results from the RNAseq data is available in Supplementary Data 1. Overall, 4559 grape genes presented significant differential expression (DE, *P < *0.05, DESeq differential expression test, *n* = 3 per treatment) as a result of *Xf* challenge, when their expression in *Xf-*inoculated vines (i.e., wild type or *wzy* mutant) was compared against that in the 1× PBS controls. DE genes that represent up-regulations were further classified into groups I to IX according to their patterns of expression (Fig. 3a). Significant correlation (*r* = 0.71, *P* = 1.37 × 10^−06,^ Pearson correlation, 9 genes, *n* = 3 per treatment) between RNAseq and qRT-PCR data was detected when we examined the expression of a subset of genes from all groups that showed upregulation in response to wild type and *wzy* cells (Supplementary Data 2). Functional pathways induced as a result of inoculations with wild type or *wzy* mutant cells were determined by enrichment analysis (*P* < 0.05, hypergeometric test, *n* = 3 per treatment) of functional categories in the groups of upregulated genes (Fig. 3b, c; Supplementary Data 3).

Group I corresponded to 200 genes significantly upregulated only in response to the *wzy* mutant at 8 h post-inoculation, but not to inoculation with wild type cells. Notably, genes associated with plant responses to biotic stresses were predominantly enriched in this group. This included the enhanced expression of several pathogenesis-related (PR) genes, including two *PR-1* precursors (*VIT_03s0088g00700, VIT_03s0088g00710*), β-1,3 glucanase (*PR-2*) (*VIT_08s0007g06040*), a class 4 chitinase (*PR-3*) (*VIT_05s0094g00330*), *PR-4* (chitinase) (*VIT_14s0081g00030*), *PR-10* (*VIT_05s0077g01530*), and *PR-10.3* (*VIT_05s0077g01530*)^31^. Additional *PR* genes included three genes involved with the production of thaumatin and a polygalacturonase-inhibiting protein, *PGIP 1* (*VIT_13s0064g01370*). The expression of genes performing antioxidant functions (e.g., thioredoxins and glutaredoxins) and ROS-scavenging enzymes (e.g., peroxidases and catalases) were also induced early in response to *wzy*. Specifically, there were three Class III peroxidases (*VIT_18s0001g06840*; *VIT_18s0001g06850*; *VIT_18s0001g06890*). These peroxidases accumulate in abundance in xylem sap during colonization by vascular pathogens^4,32^. Their functions include specific roles in defense against pathogen infection, such as enhanced production of ROS (as signal mediators and antimicrobial agents) and enhanced production of phytoalexins^33^. The upregulation of these peroxidase genes corroborates our phenotypic data of an enhanced and dynamically different production of ROS in the xylem of *wzy*-inoculated plants. Group I was also enriched in plant secondary metabolic pathways, which produce a variety of antioxidants and/or phytoalexins. There was an increase in the expression of enzymes contributing to phenylpropanoid biosynthesis, such as a stilbene synthase (*VIT_16s0100g01200*) and a chalcone reductase (*VIT_05s0077g02150*) connected with production of flavonoids. Genes associated with salicylic acid (SA)-mediated defense pathways were enriched, indicating that the *wzy* mutant is activating phytohormone signaling pathways as early as 8 h post-inoculation. In addition, the upregulation of *PR-1* genes (known markers of SA) further supports the role of SA in activating defenses when grapes have the ability to perceive *Xf* attack. These enriched pathways were strikingly absent in wild type-inoculated vines at this time point (Fig. 3b).

There were over 800 genes in group IV, which corresponded to transcripts specifically upregulated at 8 h post-inoculation in response to wild type cells. Interestingly, these genes were enriched (*P* < 0.05, hypergeometric test, *n* = 3 per treatment) primarily in responses to abiotic or general stresses (i.e., drought, oxidative, temperature, and wounding stresses) rather than biotic stresses and were not directly related to immune responses, as was observed in the *wzy* mutant-inoculated plants. Group IV included numerous genes functioning in plant cell wall modification and metabolism, including pectin esterases, polygalacturonases (PGs), expansins, and a xyloglucan endotransglucosylase/hydrolase (XTH). The upregulation of a PG-coding gene (*VIT_01s0127g00400*) at 8 h post-inoculation with the wild type strain was further validated by qRT-PCR (Supplementary Data 4). XTH enzymes are implicated in the early events of abiotic stress responses, including induction by drought and in response to the stress hormone ethylene^46,47^, whereas PGs could be involved in tylose formation by weakening the primary cell walls of xylem-associated parenchyma cells. Enrichment of genes associated with ethylene biosynthesis and signaling as well as numerous ethylene-responsive transcription factors (ERF1, ERF003, ERF011, ERF related to APETALA2) were also observed in wild type-inoculated plants 8 h post-inoculation. In the context of PD, ethylene has also been linked to tylose production, which requires extensive reorganization of plant cell walls^34^. There was also an enrichment of genes encoding components of the lignin (e.g., laccases) and suberin biosynthetic pathways that were unique to wild type-inoculated plants at this time point. Both lignin and suberin are important for wound healing and fortification of xylem walls. Suberization can also help prevent unnecessary water loss during drought stress^35^ and has been implicated in preventing lateral colonization of other vascular pathogens, such as *Verticillium* spp.^35,36^. Here we report suberin deposition in the xylem of wild type-inoculated vines, primarily associated with suberized tyloses (Supplementary Notes 1, 3, and 4; Supplementary Figure 2). Therefore, in the context of PD development, it is likely that suberization serves to mitigate unnecessary water loss from the xylem and may act as a physical barrier, attempting to block systemic spread of *Xf*. There were also genes enriched in general stress responses and responses related to dehydration, indicating that the plant is, in part, initially perceiving wild type *Xf* infection as water stress (abiotic) rather than pathogen (biotic) stress.

At 24 h post-inoculation, 164 genes were significantly upregulated specifically in response to the *wzy* mutant (group II), and there were 746 genes that showed significant upregulation in response to wild type cells at this time point (group V). Grapevines inoculated with wild type cells continued to express a number of genes related to plant cell wall modification/metabolism, including pectin esterases and expansins.

In addition to continued responses to abiotic stress, there were a large number of genes enriched in responses to biotic stress at this time point, indicating that at this later time point of 24 h, grapevines are now perceiving and responding to *Xf* as a biotic invader, as opposed to solely an abiotic stress. Interestingly, enrichment of polysaccharide metabolism pathways included two genes functioning specifically in callose biosynthesis (callose synthase, *VIT_13s0156g00210* and 1,3-beta-glucan synthase, *VIT_19s0138g00120*). This indicates that production of callose is initiated very early in the wild type infection process and supports our phenotypic data indicating a pronounced deposition of callose in the phloem of wild type-inoculated plants heavily infected with PD (Supplementary Figure 2). Enriched pathways included those specific to plant–pathogen interactions, i.e., NBS-LRR superfamily genes and secondary metabolic pathways, such as phenylpropanoid and flavonoid biosynthesis. There was consistent enrichment of genes belonging to ethylene-mediated signaling pathways, in addition to JA, auxin, and ABA signaling pathways. All of these pathways indicated above were distinctly enriched in wild type-inoculated vines and not in *wzy*-inoculated vines at the 24 h time point (Fig. 3c).

These data indicate that grapevines differentially perceive *wzy* mutant cells compared to wild type cells during the early infection process. Perception of the *wzy* mutant activates early defense networks associated with biotic stresses involved in the swift activation of PTI responses, in particular a robust production of ROS and downstream SA-mediated defense pathways that were not observed in plants inoculated with wild type *Xf*.

### Temporal and spatial dynamics of response to *Xf*

To characterize trends of gene expression over time, and in local (POI) and systemic tissue following infection by wild type and *wzy* bacteria, we performed additional RNAseq analyses on petioles collected at 48 h, 1 week, and 4 weeks post-inoculation, in addition to 1× PBS-inoculated controls. Grape genes with significant DE (*P* < 0.05, DESeq differential expression test, *n* = 3 per treatment) at distinct time points post-inoculation were determined by pairwise comparisons between each type of inoculation, time point, and tissue type (local or systemic) against the respective 1× PBS-inoculated control (Supplementary Figure 3A, B). We then defined 26 clusters based on the patterns of expression of the DE grape genes across the three time points (Supplementary Figure 3C; Supplementary Data 4). For example, C05 (cluster 5) contains DE genes that are upregulated at 48 hpi, but then have steady expression at 1 week and 4 weeks post-inoculation as compared to the 1× PBS negative control. By performing enrichment analysis in the gene clusters (*P* < 0.05, hypergeometric test, *n* = 3 per treatment), we identified over-represented functions related to plant immune responses that were distinct to each inoculation and tissue type over time (Supplementary Figure 3A, B).

Genes encoding key facets of SA-mediated signaling pathways (Enhanced disease susceptibility 1 (*EDS1*) genes, *VIT_17s0000g07370* and *VIT_17s0000g07420*) were uniquely expressed in local tissue of *wzy*-inoculated plants; these genes had steady expression at 48 h and 1 week post-inoculation and then were upregulated at 4 weeks post-inoculation (C25). EDS genes encode proteins associated with the SA pathway and have been implicated in grapevine defenses against powdery mildew^37^. Furthermore, a *PR-1* gene (*VIT_11s0052g01620*) was upregulated at 48 h post-inoculation and then steadily expressed in systemic tissue. Upregulation of SA-associated genes in both local and systemic tissues at these later time points post-inoculation with *wzy* cells is consistent with the early time points as described above. Notably, systemic induction of the SA-mediated defense pathways was not evident in wild type-inoculated plants. Instead, wild type cells induced genes associated with JA-mediated defense pathways in local tissue. This included 9 genes encoding proteins functioning in the metabolism of alpha-linolenic acid, which serves as an important precursor in the biosynthesis of JA^37,38^. These genes had steady expression at 48 h and 1 week post-inoculation and then were upregulated at 4 weeks post-inoculation (C25).

Enrichment analysis of wild type-responsive genes in systemic tissue revealed genes encoding ERF transcription factors that were upregulated at 4 weeks post-inoculation (C25), demonstrating that activation of ethylene-mediated signaling occurs throughout the infection process (early at 8 h and also later time points) in both local and systemic tissue. Stimulation of ethylene-mediated signaling may promote the prolific production of tyloses formed in wild type-inoculated vines during the infection process (Supplementary Figure 4)^12^. At 1 week post-inoculation, genes enriched in JA-mediated signaling pathways were uniquely upregulated in systemic tissue of wild type-inoculated vines, and expression continued to increase at 4 weeks post-inoculation (C19). This consistent enrichment and upregulation provides further support for the role of JA in grapevine responses to wild type *Xf*^14^. Our findings established that this phytohormone pathway is initiated within the first 24 h of the infection process and that stimulation of the JA pathway is consistently maintained in both local and systemic tissues of wild type-inoculated plants. In addition, 7 genes enriched in callose biosynthesis were uniquely upregulated at 1 week post-inoculation with wild type cells (C20), and expression remained steady at 48 h and 4 weeks post-inoculation. This is over half of the total callose-related genes in the genome. Callose deposition is an important defense mechanism in plants, and specifically for grapevine in response to the foliar powdery mildew pathogen, *Erysiphe necator*^39^, but it has not yet been implicated in *Xf* infections. As the infection progressed, we observed upregulation of nearly 60% of the total callose-related genes in the genome in systemic tissue in response to wild type cells. We also visually observed callose deposition in the phloem (Supplementary Note 1, 3, and 4; Supplementary Figure 2). The phloem is typically overlooked in the context of this xylem-limited disease, and these data clearly indicate that the phloem tissue is perceiving infection that is limited to the xylem compartment, demonstrating communication between the two tissue types. RNAseq results were validated with qRT-PCR on a select nine DE genes (Supplementary Data 2).

### Transcriptional response to purified LPSs

**Fig. 4:**
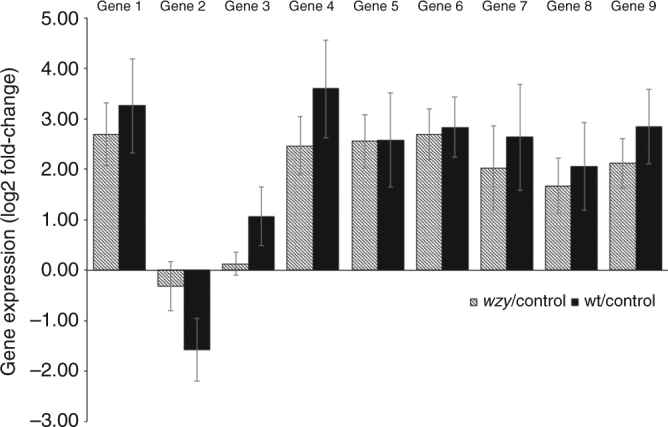
Gene expression profiles of plants inoculated with purified LPSs from wild type or the *wzy* mutant. The bar plot depicts gene expression fold-changes (log2) when compared to diH_2_O control inoculations. RNAs from duplicate petioles were pooled to generate cDNA, and the experiment was repeated twice. Genes 1–9 correspond to: *VIT_11s0052g01780* (1-deoxy-d-xylulose-5-phosphate synthase), *VIT_00s0253g00040* (monocopper oxidase), *VIT_08s0040g02200* (peroxidase ATP2a), *VIT_01s0127g00400* (polygalacturonase), *VIT_14s0060g00480* (S-adenosylmethionine synthetase 1), *VIT_13s0067g02360* (peroxidase, class III), *VIT_11s0052g01650* (pathogenesis-related protein 1 precursor), *VIT_04s0008g00420* (clavata1 receptor kinase), and *VIT_11s0052g01150* (nicotianamine synthase), respectively. Error bars represent the mean ± standard error of four pairwise comparisons (each of the two inoculated samples were compared to each of the two control samples)

To determine whether plants responded similarly to purified LPSs, as compared to the differential response observed to intact cells, we performed an in vivo transcription experiment using whole grapevine plants inoculated with purified LPSs that underwent rigorous purification (nuclease, protease treatment, dialysis, and ultracentrifugation). We inoculated vines with 2 μg per plant of either wild type or *wzy* LPSs or diH_2_O and monitored expression of nine grapevine genes (see Fig. 4 legend for genes tested) by qPCR. These genes were selected because they were differentially expressed between treatments in the RNAseq experiment and represent one gene from each of the nine RNAseq clusters. Eight of the nine genes were induced 24 h post-inoculation in response to both LPS treatments. However, induction levels were similar between the wild type and *wzy* LPS treatments, indicating the plants respond similarly to both forms of purified LPSs (Fig. 4). The results from this in vivo experiment with the purified LPS mirror the ROS burst elicited by extracted LPSs in the ex vivo leaf disc assays, where bursts were of similar amplitude and duration in discs exposed to either wild type or *wzy* LPSs. Thus, when inoculated as stand-alone molecules, both wild type and *wzy* LPSs induce similar plant responses. Although we observed differential induction of ROS bursts (Fig. 1c, d) or patterns of gene expression (Fig. 3; Supplementary Figure 3) in plants exposed to or inoculated with intact cells. From this, we deduce that (i) the core and lipid A portions are the major eliciting portions of the LPS, which would be equally exposed in *wzy* and wild type stand-alone, purified LPSs, and (ii) O-antigen sufficiently shields lipid A + core components, and possibly other surface PAMPs, in intact wild type cells.

### PD symptoms are attenuated in vines pre-treated with LPSs

**Fig. 5:**
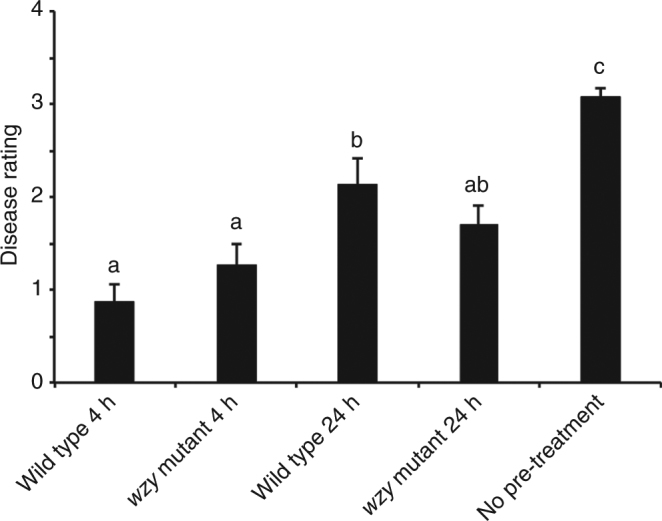
Pierce’s disease symptom severity in grapevines primed with purified *Xf* LPS. Average disease ratings of *V. vinifera* ‘Cabernet Sauvignon’ grapevines pre-treated with wild type or *wzy* LPS, or a water negative control, then challenged at 4 h or 24 h post-LPS treatment with live *Xf* cells. Disease ratings were taken at 12 weeks post-challenge. The LPS pre-treated plants are significantly attenuated in symptom development, compared with plants that did not receive pre-treatment. Graph represents the mean of 24 samples per treatment. Error bars indicate ± standard error of the mean. Treatments with different letters over the bars are statistically different (*P* < 0.05, Student’s *t*-test, *n* = 24 per treatment)

Pre-treatment of plants with LPS can prime the defense system, resulting in an enhanced response to subsequent pathogen attacks. This defense-related “memory” stimulates the plant to initiate a faster and/or stronger response against future invading pathogens^40^. In grapevine (*Vitis vinifera*), defense priming has been observed for the protection against the pathogens *Botrytis cinerea* and *Plasmopara viticola*, but these organisms have markedly different lifestyles and infection routes than *Xf*, which is limited to the xylem^41–43^. Grapevines that were pre-treated with either form of LPS (wild type or *wzy*) prior to *Xf* challenge exhibited significantly fewer PD symptoms over the course of the experiment in both the 4 and 24 h pre-treatment sample sets (*P* < 0.05, two-way non-parametric ANOVA, Mann–Whitney *U* test, *n* = 24 per treatment). Grapevines that were pre-treated for 4 h were the most attenuated in symptom development. Even after 12 weeks post-inoculation, symptoms of these 4 h plants rated only a 1 on the PD rating scale, reflecting only 1 or 2 leaves just beginning to show marginal necrosis^44^. Buffer pre-treated controls were fully symptomatic, rating ~3 on the PD rating scale (Fig. 5).

### Structural characterization of the LPS O-antigen

**Fig. 6:**
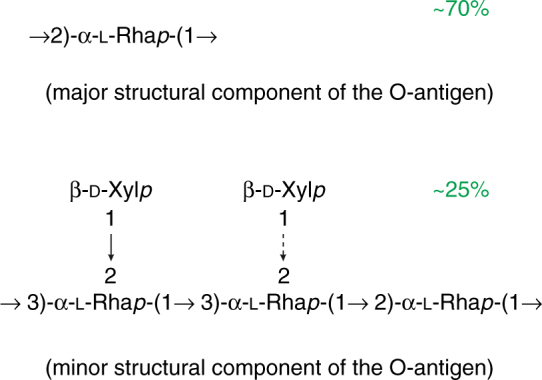
Structures of *Xf* O-antigen. Two structural components of the O-antigen were determined. The major structure (top) consists of [→2)-α-l-Rha*p*-(1→]_*n*_ homopolymer, and the minor structure (bottom) consists of [→3)-α-l-Rha*p*-(1 → 3)-α-l-Rha*p*-(1 → 2)- α-l-Rha*p*-(→]_*n*_ backbone substituted with either one or two β-d-Xyl*p* residues at C-2 of one or both of the →3)-α-l-Rha*p*-(1, residues

Compositional and structural characterization of *Xf* wild type indicated the presence of homo- and heteropolymeric O-antigen. The major O-antigen consisted of α-(1 → 2) linked-l-Rha*p* homopolymer. In addition, the second very minor heteropolymeric O-antigen backbone consisted of [→3)-α-l-Rha*p*-(1 → 3)-α-l-Rha*p*-(1 → 2)-α-l-Rha*p*-(→]_*n*_ repeats substituted with either one or two β-d-Xyl*p* residues at C-2 of either of →3)-α-l-Rha*p*-(1, respectively (Fig. 6). For a detailed description of the structural properties of O-antigen, refer to Supplementary Note 2, Supplementary Note 5, Supplementary Table 1 and Supplementary Figure 6. The *wzy*-dependent biosynthesis of O-chain was supported by the presence of high molecular mass (HMM) bands in DOC-PAGE of wild type LPS (Supplementary Figure 5A). In contrast, the *wzy* mutant produced only low molecular mass LPS devoid of polymeric O-antigen, which resolved in the gel as low molecular mass (LMM) bands (Supplementary Figure 5A). This was also supported by a lack of the high molecular mass O-antigen + core OS fraction in the *wzy*, in size exclusion chromatography (SEC) Supplementary Figure 5B. Hence, the *wzy* LPS is comprised of lipid A and core oligosaccharide, whereas wild type LPS is comprised of lipid A, core oligosaccharide and full-length O-antigen. MALDI-MS analysis of *Xf* WT and *wzy* lipid A did not indicate modification to lipid A structure (data not shown). Furthermore, based on comparative SEC (Supplementary Figure 5B) and 1D NMR (data not shown) analyses, there were no major differences observed in the core regions of the wild type and *wzy* mutant. The detailed structural characterization of the lipid A and the core is ongoing.

Although mutations in LPS can often affect the production of other cell surface polysaccharides, namely exopolysaccharide (EPS), there were no differences in colony mucoidy between the *wzy* mutant and wild type *Xf* when propagated on PD3 medium. In addition, the cells were thoroughly washed prior to the ex vivo or in vivo studies and should have little EPS associated with the cell surface at the time of application. Thus, we are confident that we can attribute the observed phenotypes, particularly at the very early time points, to the presence or lack of O-antigen and not to alterations in EPS production.

## Discussion

Studies into the biology, and particularly the plant host–pathogen interactions, of xylem-limited pathogens are challenging because they occupy a tissue compartment that is primarily non-living and not typically associated with PTI. DAB staining of petioles harvested from infected grapevines indicated that intact *wzy* cells induced a robust oxidative burst that was prominently localized to the xylem lumen, whereas wild type cells elicited a significantly muted oxidative burst in the xylem. The RNAseq data also indicated that grapevines respond markedly different to wild type cells versus *wzy* cells during the early stages of infection. Plants readily perceived *wzy* mutant cells lacking the O-antigen shield as a biotic stress to the system and activated specific defense responses (primarily those contributing to ROS production, phytoalexin biosynthesis, and *PR* gene induction) within 8 h. In addition, genes associated with SA-mediated defense pathways were enriched, indicating that the *wzy* mutant is activating phytohormone signaling pathways very early in the infection process. The upregulation of *PR-1* genes (which are known markers of SA responses) further supports the role of SA in activating defenses against the *wzy* mutant. Interestingly, consistent upregulation of SA pathway-associated genes in both local and systemic tissues at later time points in plants inoculated with the *wzy* mutant indicate that induction of SA-mediated pathways is not only maintained over time, but also systemically activated throughout the plant in response to *Xf* lacking its O-antigen. Induction of the SA-mediated defense pathway was strikingly absent in wild type-inoculated vines in local or systemic tissue at any time point we tested in this study (Fig. 3; Supplementary Figure 3). Instead, wild type cells induced genes associated with JA-mediated defense pathways early in the infection process in local tissue. These JA pathway-related genes continued to be maintained over time and were systemically activated in the plant in response to wild type *Xf*.

Wild type cells predominantly elicited responses related to abiotic stresses beginning very early in the infection process. Specifically, there was induction of a wide range of genes whose products are known to function in drought stress tolerance, including the induction of abscisic acid (ABA) biosynthetic genes, an important mediator of water-stress adaptation and suberin deposition^45^. PD-infected vines produce more ethylene than healthy vines, and exposing grapevines to ethylene mimicked a hallmark plant response associated with *Xf* infection, tylose production, which requires extensive plasticity and reorganization of the plant cell wall linked to CWDEs^34^. It is speculated that *Xf*-induced ethylene is linked to the signaling process that initiates the formation of tyloses, which exacerbates PD symptoms^12^. The concomitant upregulation of cell wall modification enzymes and ethylene biosynthetic genes as early as 8 h post-inoculation in wild type-inoculated plants indicates that the transcriptional cascade associated with tylose production initiates very early in the infection process, whereas phenotypic evidence of abundant tylose production occurs later in the infection process (Fig. 3; Supplementary Figure 3)^48^. Tylose over-production is linked to exacerbating PD symptom severity, so understanding when that process initiates could provide clues about the temporal dynamics of tylose formation and a potential path to mitigating PD at the genomic level.

Stand alone, purified LPS molecules from either wild type or *wzy* cells induced ROS bursts of similar amplitude from excised leaf discs. To mimic natural infection conditions, we also delivered purified LPSs directly to the xylem through needle inoculation into intact plants. Much like the leaf disc ROS assays, we observed similar responses to both forms of LPS. Specifically, both wild type and *wzy* purified LPSs induced gene expression to similar levels when quantifying enriched genes that were reported in the RNAseq study. Pre-treatment of plants with purified elicitors, such as LPS, can potentiate the plant defense system, resulting in tolerance to subsequent pathogen attack^49,50^. This process, known as defense priming, stimulates the plant to initiate a faster and/or more aggressive response against future invading pathogens^49,50^. Priming has been described primarily in the context of tolerance to foliar pathogens, but here we demonstrate that the primed state can be induced by pre-treating grapevines with stand-alone *Xf* LPSs, thus conferring tolerance to the xylem-limited bacterium, *Xf*. The primed state in grapevines occurred regardless if plants were pre-treated with purified LPS originating from wild type or *wzy* mutant *Xf*.

We report that the major structural component of the LPS O-antigen of *Xf* consists primarily of α-(1 → 2) linked-l-Rha*p*, and the second minor structural component of the O-antigen consists of [→3)-α-l-Rha*p*-(1 → 3)-α-l-Rha*p*-(1 → 2)- α-l-Rha*p*-(→]_*n*_ substituted with either one or two β-d-Xyl*p* residues at C-2 of one or both of the →3)-α-l-Rha*p*-(1, residues. The heterogeneity of O-polysaccharides (OPS), or the presence of different O-antigen units in the same strain, is not uncommon in phytopathogenic members of *Pseudomonadales* and *Xanthomonadales*. The lack of exacting regularity is due to the presence of a monosaccharide in nonstoichiometric amounts on the side chain or unsystematic biosynthetic polymerization^51–56^. Some strains of *Pseudomonas syringae* possess O-antigens with a repeat unit capped by additional structural domain or modified side chain^57,58^. It was suggested that modified O-repeats are linked to the non-reducing end of the OPS to terminate the polymer elongation^57^. Therefore, it is possible that the O-antigen of *Xf* is primarily comprised of  α-(1 → 2) linked-l-Rha*p* polysaccharide terminated by the capping sequence of [→2)- α-l-Rha*p*-(1 → 3)-α-l-Rha*p*-(1 → 3)-α-l-Rha*p*-(1]_*n*_ substituted with either one or two β-d-Xyl*p* residues at C-2 of one or both of the →3)-α-l-Rha*p*-(1, residues. Many phytopathogens like *Pseudomonas*, *Xanthomonas*, *Erwinia* and *Pantoea *spp. express O-chain repeating units with the backbone enriched in α or β-rhamnose residues, either in l- or d-configuration. These tri- or tetra-saccharide repeat units are often substituted with an additional 1–2 monosaccharides^59^. For example, the O-chains consisting of the [→3)-α-Rha*p*-(1 → 3)-α-Rha*p*-(1 → 2)-α-Rha*p*-(→]_*n*_ repeat motif were found in *Pseudomonas syringae*^60^ and in members of *Xanthomonas campestris*^59^. Similar to what we report in the minor O-chain polysaccharide of *Xf*, the repeat unit of O-antigen in *X. campestris* pv. begoniae GSPB 525 consists of a nonstoichiometrically xylosylated trisaccharide repeat^61^. The main α-(1 → 2) linked-l-Rha homopolymeric O-antigen discovered in *Xf* (this work) resembles the O-chain composed of β-(1 → 2) linked-L-Rha, expressed by *P. syringae* pv. mori NCPPB 1656^51^. In future work, we will elucidate the structure of the lipid A + core, a first step to identifying the elicitor portions of the LPS PAMP, and which will aid in identifying a potential receptor in grapevine.

Infection processes are dynamic, and the mechanisms by which a given bacterial species interfaces with the host immune system are multi-faceted. Currently, there are no known *Xf* secreted effectors that are involved in plant host immune recognition or those that dampen the immune response for the *Xf* pathosystem. O-antigens are the outermost exposed portion of the LPS molecule, and previous work utilizing molecular modeling has theorized that the O-antigens are not strictly rigid but can have conformational flexibility, allowing them to bend so that the polymer covers, or lies over, other adjacent surface-bound molecules^62^. It is possible that the long chain *Xf* O-antigen can take on a bent conformation on the cell surface and shields not only the LPS core + lipid A elicitor but potentially other cell surface PAMPs as well. Different subspecies of *Xf* exhibit host specificity, and in many scenarios, can colonize plants as commensals rather than acting as pathogens^10^. It is tempting to speculate that O-antigen composition has a role in host range and/or dictates the type of symbiotic association with the plant-commensalism vs. parasitism.

## Methods

### Bacterial strains and growth conditions

We used wild type *Xf* subsp. fastidiosa strain Temecula1^63^ and a *wzy* mutant strain^21^. *Xf* wild type and *wzy* mutant strains were grown for 7 days at 28 °C on solid PD3 medium.

### Purification of LPS and core-O-specific polysaccharide

LPSs were purified in the following manner for in planta gene induction (qPCR) studies, ROS induction assays and structural analyses. LPSs were isolated from *Xf* wild type and *wzy* mutant bacterial cell pellets via the hot phenol-water extraction procedure^64^. Dialyzed phenol and water phases (3500 MWCO) were freeze-dried and washed with 9:1 (v/v) ethanol in water at 4 °C. Nucleic acids and proteins were removed by overnight treatment with RNase and DNase (37 °C), followed by overnight incubation with Proteinase K (37 °C) and dialysis (2000 MWCO) at 4 °C against several exchanges of dH_2_O. Dialysate of LPS was ultracentrifuged at 100,000×*g* at 4 °C for 18 h and recovered from the pellet. LPS was dissolved in 0.1 M EDTA H4/0.3 M TEA (pH 7) and resolved on Superose 12 10/300 GL (GE Healthcare Life Sciences) FPLC column assembled with AKTA system (Amersham Biosciences) with 50 mM ammonium acetate buffer (pH 6.7) used as eluent with 0.5 mL/min flow. Eluted fractions were recorded with a refractive index (RID-10A, Shimadzu) and multiple wavelength (*λ* = 210, 254, and 280 nm; AKTA system) detectors, respectively. LPS was resolved on 18% acrylamide using deoxycholic acid (DOC) detergent^65^, followed by silver staining using the Bio-Rad Silver Staining Kit (Bio-Rad). Optionally, the gel was stained with alcian blue^66^, followed by silver staining. Void volume LPS fraction was used for O-chain separation and comparative chemical and structural studies. Carbohydrate moiety of LPS was liberated from lipid A by 1.5 h mild hydrolysis in 1% HOAc (v/v) at 100 °C and recovered from supernatant after 20 min centrifugation at 5000×*g*, at 30 °C. Similarly to LPS, free O-antigen + core moiety was dissolved in 50 mM ammonium acetate buffer (pH 6.7) and chromatographed on Superose 12 10/300 GL (see description above). Fractions were collected based on response from refractive index detector. OPS polysaccharides were hydrolyzed with 2 M TFA (120 °C, 2 h) and converted to alditol acetates (AAs) by conventional methods^67,68^. Glycosyl linkage of OPS was determined after hydrolysis with 2 M TFA at 121 °C, overnight reduction with NaBD_4_ and conversion to partially methylated alditol acetates (PMAA)^67^. Detailed GLC–MS analyses and 1D and 2D nuclear magnetic resonance spectroscopy were performed as described in the supplementary material (Supplementary Note 2 and 5).

### LPS extractions for kinetic ROS assays and defense priming

LPS extractions were performed based on the method of Marolda et al.^69^, with some modification. Briefly, *Xf* cells were grown on solid PD3 medium for 7 days at 28 °C. Cells were harvested with 1× PBS buffer and spun down to form dense pellets and stored at −80 °C. Cell pellets were washed two times with 1× PBS buffer and suspended in 300 μL Solution A (0.05 M Na_2_HPO_4_ × 7H_2_O, 0.005 M EDTA; pH 7) + 40 μL Proteinase K (Qiagen #19131). Suspensions were incubated overnight at room temperature. LPS was extracted from cell pellets using a hot phenol/water method, and resulting LPS was further purified using dialysis (MWCO 1 kD). Following purification, LPS was quantified using the Purpald Assay^70^.

All LPS concentrations mentioned throughout the manuscript were normalized to μg/mL of Kdo content using the Purpald assay and reported concentrations represent μg/mL of Kdo content or μg of Kdo content.

### Ex vivo ROS assays

Kinetic ROS assays: Leaf discs (1/8″) were punched from *Vitis vinifera* ‘Cabernet Sauvignon’ grapevines. Discs were immediately placed into individual wells of a 96-well microtiter plate (Costar catalog #3912), with each well containing 200 μL diH_2_O. Discs were allowed to sit overnight at room temperature, in the dark. The following day the diH_2_O was removed and 200 μL reactions were performed within each well. Reactions contained the following: 2 μL of a 100 nM stock of luminol, 1 μL of a 1 mg/100 μL stock of horseradish peroxidase, 177 μL of diH_2_O, and 20 μL of a 10^8^ CFU/mL suspension of *Xf* cells or 20 μL of purified LPS elicitors (at a final concentration of 50 μg/mL, which equals 10 μg of LPS (based on Kdo content) in each 200 μL reaction). Reagents were first prepared as a master mix in a Falcon tube in the following order: diH_2_O, LPS or *Xf* cells, luminol, and HRP. The ingredients were gently mixed and then, using a multichannel pipette, immediately dispensed into the wells containing the leaf discs. Water or 1× PBS negative controls were used for LPS or cell suspensions, respectively. Relative luminescence was measured in a Tecan Infinite F200 plate reader. Water or 1× PBS negative control background readings were subtracted for LPS or cell suspensions, respectively. Assays were repeated in triplicate and consisted of a total of 24 replicates/treatment. Total ROS production was determined by calculating the area under the curve for each treatment, and data were analyzed using the Kruskal–Wallis test for non-parametric one-way ANOVA. Prior to the test, data were checked for normality and homogeneity of variance and did not meet these criteria^71^. Post hoc comparisons amongst the treatments used Mann–Whitney *U* tests at *P* = 0.05.

End-point ROS production assay: Leaf discs were prepared as described above. A total of 100 μL reactions were performed within each well of a black 96-well plate (Costar, Corning, NY). Purified LPSs (wild type or *wzy*) were sonicated for 20 s and then diluted to the appropriate concentrations to deliver 5, 10, 12.5, or 20 μg (based on Kdo content) of LPS in 50 μL of diH_2_O in a final reaction volume of 100 μl. ROS (hydrogen peroxide) was quantified using the Amplex Red Hydrogen Peroxide detection assay kit (ThermoFisher Scientific, Waltham, MA). Assay reagents were prepared according to the manufacturer’s protocol as a master mix in the following order: 1× reaction buffer, Amplex Red, and HRP. The diluted purified LPSs were sonicated again for 20 s and then added to the master mix at the appropriate concentrations. The ingredients were gently mixed and then, using a multichannel pipette, immediately dispensed into the wells containing the leaf discs. Negative control wells contained water in place of the LPS in the reaction mixture. Relative fluorescence was measured in a Tecan Infinite F200 plate reader. The negative water control background readings were subtracted from all samples, and an internal hydrogen peroxide standard was included on all plates to normalize the data across plates. The data represent RFU values collected from five biological replicates. Two-way analysis of variance (ANOVA) was run using the GLM procedure in SAS 9.3 from SAS Institute, Inc. to test the effect of treatment and concentrations on ROS production. Further multiple comparisons were conducted using Tukey adjustment to determine if ROS levels are significantly different from one another, *P* = 0.05.

### In situ localization of *Xf*-induced H_2_O_2_

This procedure was adapted from the methods of Liu et al.^71^ Nitrocellulose membrane (0.45 μm in pore size) was soaked in 5 mg/mL DAB-HCL solution (pH 3.8) and then dried at room temperature for 30 min in the dark. Tissue printing was performed at ~ 20 °C. At 15 min post-inoculation with wild type *Xf, wzy* cells (40 μL of 10^8^ CFU/mL suspension), or a 1× PBS buffer control, petioles were removed, and freehand sections were made of ~1 mm thickness using a razor blade (completed within 5 s). The sections were gently pressed onto the impregnated nitrocellulose membrane for 10 s. The membrane was washed with 100% EtOH to remove any possible inhibitors and photographed under a stereomicroscope (M165C, Leica Microsystems CMS GmbH, Wetzlar, Germany) after 5 min at room temperature. Assays were repeated in triplicate and consisted of a total of 9 replicates per treatment. Image analysis was conducted using ImageJ according to the methods of Bunderson et al.^72^. Briefly, the DAB-stained images were converted to grayscale and then minimum and maximum threshold values were established to remove background staining. Mean gray values for each treatment were calculated and analyzed by one-way ANOVA. Data were checked for normality and homogeneity of variance and met these criteria. Post hoc comparisons amongst the treatments used Tukey’s honestly significant difference (HSD) test at *P* = 0.05.

### H_2_O_2_ survival assay

*Xf* wild type or *wzy* cells were tested for survival under oxidative stress (H_2_O_2_). *Xf* wild type or *wzy* mutant cells were grown on solid PD3 medium for 7 days at 28 °C. Cells were harvested with 1× PBS buffer and adjusted to OD_600_ 0.25 (10^8^ CFU/mL). Treatments consisted of 500 μL of cell suspension and 500 μL of 1× PBS, with or without the addition of 5 μL of H_2_O_2_ for a final concentration of 100 μM. Suspensions were incubated at 28 °C at 100 rpm for 10 min and then immediately placed on ice. The resulting suspension was diluted and plated onto solid PD3 medium according to standard methods. Assays were repeated in triplicate, using freshly harvested *Xf* cells for each replication. Percent survival was determined as the number of CFU/mL in treated cells divided by the number of CFU/mL in untreated cells (without H_2_O_2_), multiplied by 100. Prior to the test, data were checked for normality and homogeneity of variance and did not meet these criteria. Comparisons amongst the treatments used Mann–Whitney *U* tests at *P* = 0.05.

### Transcriptional profiling of grapevine responses to *Xf*

For the RNAseq experiments, *Vitis vinifera* ‘Cabernet Sauvignon’ grapevines were needle inoculated with either *Xf* wild type, *wzy* cells, or 1× PBS buffer as previously described^21^. Fifteen plants per treatment were inoculated per time point. One petiole per plant was harvested at 8 and 24 h post-inoculation at the point of inoculation. The RNA was pooled from five replicate petioles from each treatment to build three biological replicate cDNA libraries for each treatment. The same experimental design was applied to the 48 h, 1 week, and 4-week time points on a new set of plants. Local and systemic petioles were harvested at each of those time points, and RNA from five petioles were pooled to construct cDNA libraries to make three biological replicate cDNA libraries per local and systemic treatment.

Following harvest, petioles were immediately placed into liquid nitrogen and stored at −80 °C until RNA extraction. The frozen tissue was ground to a fine powder using stainless steel beads in a TissueLyser II (QIAGEN Inc., USA) for 30 s with a frequency of 30 per s. Total RNA was extracted from 0.1 g of ground tissue per sample. The frozen tissue was homogenized by inversion in 500 µl of extraction buffer (PureLink Plant RNA Reagent, Ambion cat# 12322-012) for 5 min at room temperature. After homogenization, 100 µL of Plant RNA isolation Aid (Ambion cat# AM9690) was added to the suspension and mixed by inversion for 5 min at room temperature. Samples were centrifuged at top speed for 5 min at room temperature to pellet insoluble debris and other contaminants. The supernatant was transferred to a new tube and then 100 µL of 5 M NaCl and 300 µL chloroform were added and mixed for 5 min by inversion. Samples were centrifuged at 12,000×*g* for 10 min at 4 °C to separate the phases, and the upper aqueous phase was transferred to a new tube. Samples were treated with DNase RNase-free (5 U) and incubated for 20 min at 37 °C. After DNase treatment, 500 µL of chloroform was added and mixed for 5 min as described above. To separate the phases, the samples were centrifuged again at 12,000×*g* for 10 min at 4 °C, and the upper aqueous phase was transferred to a new tube. An equal volume of isopropyl alcohol was added, after the samples were mixed by inversion and incubated at room temperature for 10 min. Precipitated RNA was centrifuged at 12,000×*g* for 10 min at 4 °C, and the supernatant was discarded. The RNA pellets were washed with 1 mL of 80% ethanol and centrifuged at 12,000×*g* for 2 min at room temperature. The pellets were then air-dried at room temperature and re-suspended in 30 µL RNase-free water. RNA quality was evaluated by gel electrophoresis, and quantity was determined using the Qubit fluorometer. cDNA libraries were generated from mRNA and sequenced using an Illumina HiSeq 3000 platform. The sequencing of four HiSeq lanes generated a total of 763 million 50 bp single-end reads. Reads were trimmed and filtered to retain high-quality sequence information only (*Q* > 20). An average of 24.5 ± 3 millions of reads per sample (87.6 ± 1.7% of the total) were unambiguously mapped on the reference PN40024 transcriptome. Counts were normalized to control for technical variation using DESeq2^73^, which was also used for statistical testing. Functional annotations and biological processes associated to the differentially expressed grape genes were obtained from VitisNet (https://www.sdstate.edu/ps/research/vitis/pathways.cfm)^74^. Enrichment analyses of grape biological processes were performed in R using a hypergeometric test with a significant cut-off of *P* < 0.05. For all the RNAseq studies, we did not have any expectation on effect size as this was the first time *Xf* impact on transcriptome at these early time points has been measured. Therefore, we did not have effect size and variance information to calculate power and sampling size.

### Validation of RNAseq data

For gene induction studies following inoculation with purified LPSs and RNAseq validation experiments, the following conditions were used. cDNA was synthesized from 500 ng of total RNA using M-MLV Reverse Transcriptase (Promega). qRT-PCR was performed on a StepOnePlus PCR System using Fast SYBR Green Master Mix (Applied Biosystems). The qRT-PCR conditions were as follows: 95 °C for 10 min, followed by 40 cycles of 95 °C for 3 s and 60 °C for 30 s. *VvActin* (*VIT_04s0044g00580)* (106), was selected as a reference gene to linearize the transcript levels for all genes of interest using the formula 2^(Reference gene CT − Gene of interest CT)^^75^. Three biological replicates were tested per inoculated *Xf* strain (wild type and *wzy* mutant) and each of the time points. The specificity was confirmed by analyzing the melting curves at temperatures ranging from 60 to 95 °C. The primer sequences can be found in Supplementary Data 5.

### Gene expression following inoculation with purified LPSs

Plants were needle inoculated with 40 µL of a 50 µg/mL stock solution of wild type or *wzy* LPS, equal to 2 µg (based on Kdo content) of LPS inoculated into each plant. The LPS used was purified as described in the “Purification of LPS and core-O-specific polysaccharide” section. The nearest petiole was harvested 24 h post-inoculation. Petioles were immediately frozen in liquid nitrogen, and RNA was extracted as described above. cDNA was generated and qPCR carried out as described above. Statistical differences between transcriptional changes induced by *wzy* and wild type *Xf* cells were tested using a two-tailed Student’s *t*-test. Log2 transformed fold-changes relative to expression in water-treated control samples were used for statistical testing.

### Defense priming experiments

A total of 24 plants were needle inoculated with 40 µL of a 50 µg/mL stock solution of wild type or *wzy* extracted LPS, equal to 2 µg (based on Kdo content) of LPS inoculated into each plant followed 4 or 24 h later with needle inoculation of 40 µL of a 10^8^ CFU/mL inoculum preparation of *Xf* wild type cells. Vines that received 1× PBS buffer only, prior to inoculation with *Xf* wild type cells, served as the controls. All vines were randomized in the greenhouse and PD symptom progression was assessed according to the standard PD rating scale of 0–5^44^.

### Data availability

The Illumina sequencing reads can be accessed in the National Center for Biotechnology Information’s Gene Expression Omnibus (GEO) under the accession number GSE87643 (http://www.ncbi.nlm.nih.gov/geo/query/acc.cgi?acc=GSE87643). The authors declare that all other data supporting the findings of this study are included in the main manuscript or Supplementary Information files or is available from the corresponding author upon request.

## Electronic supplementary material

Supplementary Information

Description of Additional Supplementary Files

Supplementary Data 1

Supplementary Data 2

Supplementary Data 3

Supplementary Data 4

Supplementary Data 5
