# Supplementary material for: Lipopolysaccharide O-antigen delays plant innate immune recognition of Xylella fastidiosa
Source: Nat Commun. 2018 Jan 26;9:390. doi: 10.1038/s41467-018-02861-5 (PMC5786101; doi:10.1038/s41467-018-02861-5)
Supplement: Supplementary file 1 — Supplementary Information [file 41467_2018_2861_MOESM1_ESM.pdf]

Supplementary Note 1. Late infection stage phenotypes in wild type infections

The plant vascular tissue can initiate reinforcement of the cell walls to limit bacterial growth in infected plants. This includes callose and suberin deposition[1]. Because the RNASeq studies revealed hallmarks of callose and suberin deposition in wild type-inoculated plants that were absent in *wzy* mutants very early in the infection process, and were maintained over time, we sought to determine if callose and suberin deposition were associated with xylem-limited *Xf* infections. These two defenses have not been described at the phenotypic level for *Xf*. Indeed, light microscopy of infected stems revealed widespread deposition of callose in the phloem tissue of *Xf* wild type-infected plants (Supplementary Figure 2, arrow), suggesting that there is communication between the xylem and phloem regarding the presence of *Xf*. In addition, there was a pronounced deposition of suberin associated specifically with tylose-occluded vessels (Supplementary Figure 2, *). In contrast, *wzy* mutant-infected plants showed little to no evidence of either callose or suberin deposition in the vascular tissue, and cross-sections of these plants looked similar to cross sections of 1X PBS control plants.

Tyloses are vascular occlusions that are outgrowths of the xylem parenchyma cells into the vessel lumen. These are commonly initiated by *Xf* infection. Tylose formation occurs later in the infection process, and the abundant tyloses observed in PD-infected vines exacerbate symptoms[2]. We examined PD symptoms and tylose formation in grapevines at 18 weeks post-inoculation with wild type or *wzy* mutant *Xf* cells, compared with 1X PBS control vines. *Wzy* mutant-inoculated vines rated a 2 or below, representing a few leaves exhibiting marginal necrosis; wild type-inoculated vines rated over 3, representing over half of the vine exhibiting

foliar necrosis; and 1X PBS controls rated 0, showing no PD symptoms (Supplementary Fig 4 panel A). We observed marked differences in the abundance of tyloses in response to wild type vs. *wzy* mutant-inoculated plants. In wild type-inoculated vines, tyloses were present in nearly all xylem vessels (Supplementary Fig 4 panel B), and vessels were often completely occluded with multiple tyloses (Supplementary Fig 4 panel C). In contrast, *wzy* mutant-inoculated vines contained very few tyloses. In the case where a tylose was present, it was often one tylose that only partially occluded the vessel. All control vines, inoculated with 1X PBS, were free of occlusions.

Supplementary Note 2. Characterization of the LPS O-antigen shield

DOC-PAGE analysis of purified LPS indicated a high molecular weight O-antigen in wild type LPS (SupplementaryFigure 5 A, lane 1, arrow). In contrast, in the *wzy* mutant LPS (Supplementary Figure 5 A, lane 2), the HMM O-antigen band was absent, and the lower bands on the gel were more pronounced, indicative of reduced or no O-antigen production. This observation was consistent with chemical analysis and subsequent structural analysis. Wild type LPS was primarily composed of rhamnose (Rha) and xylose (Xyl). In addition, we identified mannose (Man), glucose (Glc), galacturonic acid (GalA), 3-deoxy-D-*manno*-2-octulosonic acid (Kdo), glucosamine (GlcN), trace (< 0.1 mol percent) of 2-methyl -rhamnose (Supplementary Table 1) and hydroxylated, 10:0(3OH), and 12:0(3OH), f. In the *wzy* mutant, the amounts of Rha and Xyl were reduced, relative to the other glycosyl LPS constituents, e.g. an increased amount of GlcN, GalA and Glc, and 2-methyl-rhamnose was not detected (Supplementary Table 1).

There was also a higher level of hydroxylated fatty acids (not shown) compared to the wild type (not shown). Therefore, the DOC-PAGE and composition analyses show that the wild type O-antigen is likely a polymer composed of rhamnose and xylose, whereas in the *wzy* mutant, this polymer is severely truncated.

To determine the structure of the O-antigen, the polysaccharide moiety of LPS (O-antigen + core) was liberated from lipid A and resolved based on molecular size by using size exclusion chromatography (SEC) (Supplementary Figure 5 B). Wild-type LPS produced largely a polysaccharide, Fraction III (40.8% total column load, average molecular mass of approximately 10-20kD), and a mixture of oligosaccharides (24.8% of total column load) in Fraction IV. In comparison, a majority of *wzy* LPS consisted of the mixture of Fraction IV oligosaccharides (55.0% of the column load, average molecular mass below 10kDa). The Fraction IV oligosaccharides likely represent a mixture of different core oligosaccharides with and without attached truncated O-antigen oligosaccharides. Fraction I, that eluted at the void volume (Vo) of the column, contained traces of intact LPS due to incomplete mild-acid hydrolysis. The O-antigen present in Fraction III was isolated and subjected to further analysis.

Monosaccharide analysis, including the determination of absolute configurations of the wild type strain O-antigen and methylation analysis, confirmed the presence of L-rhamnose and D-xylose in an 9:1 molar ratio with (8%) terminal Xyl*p*, (76%) 2-substituted Rha*p*, (3%) 3-substituted Rha*p*, and (8%) 2,3-disubstituted Rha*p*. In addition, ^{1}H and ^{13}C NMR analyses revealed two series of anomeric signals with a ratio of integral intensities ~ 4:1 (Supplementary Figure 6 A), indicative that the O-antigen contains two structural features (Fig 6).

1D ^{1}H NMR spectrum showed six minor anomeric signals of equal intensity in the anomeric region (δ 4.4-5.2), named from **A** to **G**, according to decreasing chemical shifts values, and one major signal named **H** (Supplementary Figure 6, Supplementary Table 2). The δ values of residues **A**, **B**, **C**, **D**, **E** and **H** suggested the presence of α-configurations (singlets), and the δ values of residues of **F** and **G** indicated the occurrence of β-configurations (broadened doublets, $J_{1,2}$ ~8). The spectrum presented one broad high-filed signal at ~ δ 1.30, arising from the methyl groups of Rha residues. The complete assignments of the ^{1}H and ^{13}C chemical shifts of the O-antigen were possible based on 2D TOCSY (mixing times, 30-150 ms), NOESY (200 ms), and HSQC experiments (Supplementary Table 2). Comparison of TOCSY spectra with increasing mixing time allowed the assignments of sequential order of the chemical shifts belonging to the same spin system. Residues **A**, **B**, **C**, **D**, **E** and **H**, with short H-1 tracks (only H-2 is seen) and the typical H-6 signals for 6-deoxyhexoses, represent the Rha residues. The complete spin system of residues **F** and **G** was indicative of a *xylo*-configuration. In addition, the 2D TOCSY (150 ms), NOESY (200 ms) and ^{1}H-^{13}C HSQC spectra showed the predominant signals for **H** residue (Supplementary Figure 6 A,B,C respectively). The TOCSY **H** H-1 track (δ 5.10) showed cross-peak with **H** H-2, whereas the resonances for **H** H-2,3,4,CH_3 were found via H-2 track (Fig S6 A). The ^{13}C NMR data, demonstrated a downfield position of **H** C-2 at δ 79.3, suggesting 2-substituted α-Rha*p*[3] (Fig S6 C). The NOESY inter-residue cross-peak of **H** H-1, with another **H** H-2, indicated a **H**(1-2)**H** linkage (Supplementary Figure 6 B). Based on methylation analysis and 1D/2D NMR data, the major structural component present in Fraction III (~70%) was a linear rhamnan with the following structure:

→2)-α-L-Rha*p*-(1→
H

In addition to the homopolymer NMR signals (residues **H**), five minor anomeric signals belonging to α-Rha*p* (residues **A**-**E**) and β-Xyl*p* (residues **F** and **G**) were identified. Residues **A** and **C** were assigned to 2-substitituted α-Rha*p* (downfield shift of **A** C-2 at δ 79.3 and **C** C-2 δ 79.6), and the residue **D** was identified as 3-substitituted α-Rha*p* (downfield shift of **D** C-3 at δ 76.2). The signals for C-2 (δ 79.6) and C-3 (δ 76.2) of residues **B** and **E** were both shifted downfield indicating the presence of 2,3-disubstituted α-Rha*p* (Supplementary Figure 6 C). ^{13}C chemical shifts of residues **G** and **F** were consistent with terminal β-Xyl*p*[3]. The 2D NOESY inter-residue cross-peaks (Supplementary Figure 6 B) allowed assignment of the glycosyl sequence of the second polymer. In particular, 2-substitution of **A** and **C** residues was reflected by **D** H-1/**A** H-2 and **E** H-1/**C** H-2, cross-peaks, respectively. Furthermore, NOE cross-peaks between **C** H-1/ **B** H-3, **A**1 H-1/**B** H3, **G** H-1/**B** H-2 and **B** H-1/ **E** H-3, **F** H-1/**E** H-2 provided information about the location of branching positions of residues **B** and **E**. The 3-substitution of residue **D** was observed by the inter-residue connectivity of **B** H-1/**D** H-3. The absence of downfield glycosylation shift at C-2 carbon of residue **D** (δ 69.7) supports the occurrence of 3-substituted residue **D** (Supplementary Figure S6 C). Combining all analytical data, a minor component present in Fraction III (~25%) consisted of -α-L-rhamnan backbone substituted with either two or one β-D-Xyl residues:

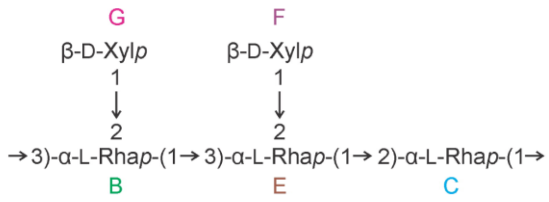

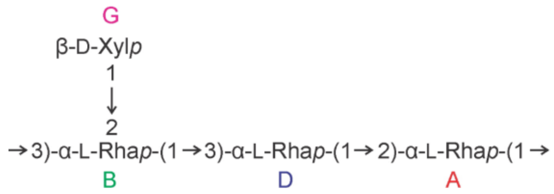

In conclusion, we identified two structural components of the O-antigen. The major component consists of α-(1→2) linked-L-Rha*p* homopolymer. The second minor structural component of O-antigen consists of [→3)-α-L-Rha*p*-(1 →3)-α-L-Rha*p*-(1 →2)-α-L-Rha*p*-(→]$_n$ substituted with either one or two β-D-Xyl*p* residues at C-2 of one or both of the →3)-α-L-Rha*p*-(1, residues (Fig 6). In contrast the *wzy* mutant LPS is devoid of O-antigen polysaccharide (Supplementary Figure 5 A,B).

Supplementary Note 3. Tylose development in PD-infected grapevines.

Stem sections of *Vitis vinifera* 'Cabernet Sauvignon' were harvested at 18 weeks post-inoculation with *Xf* wild type, *wzy* mutant, or a 1X PBS negative control. Tissue was fixed in 80% ethanol prior to histological examination. Freehand sections were made of approximately 100 µm, stained with Toluidine Blue O (0.05%), and observed using a brightfield microscope (DM4000, Leica Microsystems CMS GmbH, Wetzlar, Germany).

Supplementary Note 4. Suberin and callose deposition in PD-infected grapevines.

Stem sections of *Vitis vinifera* ‘Cabernet Sauvignon’ were harvested at 18 weeks post-inoculation with *Xf* wild type, *wzy* mutant, or a 1X PBS negative control. Tissue was fixed in 80% ethanol prior to histological examination. Freehand sections were made of approximately 100 μm and stained with Sudan III (0.2%), followed by Aniline Blue (0.05%), and observed using a brightfield microscope (DM4000, Leica Microsystems CMS GmbH, Wetzlar, Germany).

Supplementary Note 5. Structural characterization of the LPS O-antigen

The glycosyl and fatty acid composition of the LPS was determined by the preparation of trimethylsilyl (TMS) methyl glycosides and after methanolysis with 1M methanolic HCl at 80 ºC for 18 h, in the presence of an internal standard of inositol, and analyzed by GLC-MS (93, 94). Obtained derivatives were analyzed on Hewlett-Packard HP5890 gas chromatograph equipped with mass selective detector 5970 MSD using EC-1 fused silica capillary column (30m · 0.25 mm I.D.) and the following temperature program: 80ºC for 2 min, then increased to 160ºC at 20ºC/min, and to 200ºC at 2ºC/min followed by an increase to 250 °C at 10°C/min with an 11 min hold.

GLC-MS analysis of alditol acetates was performed on an HP-5890 GC interfaced to a mass selective detector 5970 MSD using a Supelco SP2330 (30m · 0.25 mm ID) capillary column with temperature program: 80ºC for 2 min, then increased to 170ºC at 30ºC/min, and to 235ºC at 4ºC/min with a 20 min hold. Absolute configuration of O-chain monosaccharides were determined by comparative GLC analysis of the trimethylsilylated (-)-2-butyl glycosides with

that of authentic monosaccharide standards [4,5] using an Equity-1 fused silica capillary column and GC-MS condition similar with analysis of TMS methyl glycosides.

Supplementary Note 6. 1D and 2D nuclear magnetic resonance spectroscopy

Polysaccharide samples were exchanged twice with 99.9% D_2O and finally dissolved in 100% D_2O (Cambridge Isotope Laboratories, Andover, MA) to a final concentration of approximately 2.5 mg/mL. 1D proton and 2D (^1H -^1H TOCSY, ^1H-^{13}C-HSQC, -HMBC) spectra were acquired at 25°C with standard “Presat” solvent signal suppression on a Varian 600 MHz spectrometer equipped with a 3 mm cold probe (Varian, Inova Palo Alto, CA). The NMR acquisitions were processed using MNova software (Mestrelab Research, Santiago de Compostela, Spain). The spectra were referenced relative to the DSS signal (™$_H$=0 ppm; ™$_C$=0 ppm).

SUPPLEMENTARY FIGURES

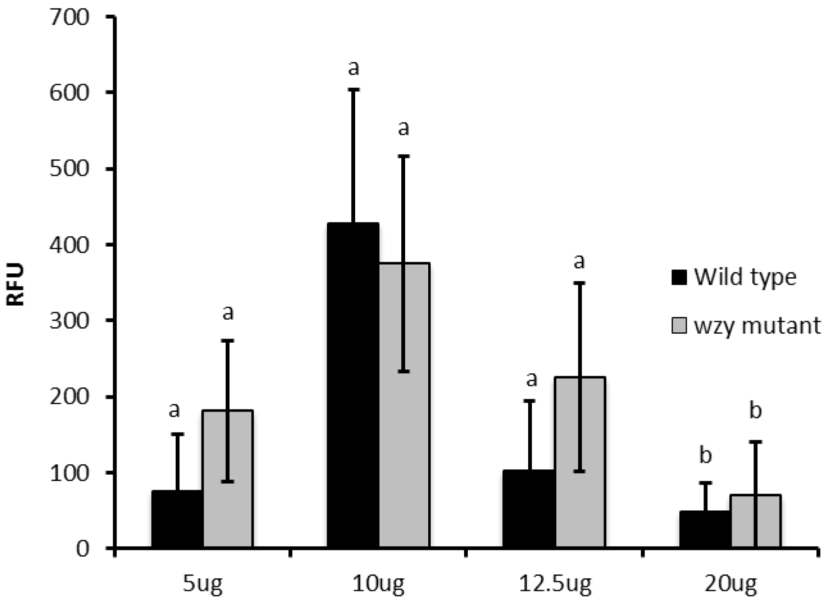

Supplementary Figure 1. LPS-modulated ROS production by purified LPSs *ex vivo*. Discs of *V. vinifera* 'Cabernet Sauvignon' leaves were treated with 50 µL containing 5, 10, 12.5 or 20 µg wild type or *wzy* purified LPS suspended in diH_20, or diH_2O alone. **(A)** The amount of ROS production was similar for both wild type and *wzy* LPSs. Total ROS production is reported as RFUs (relative fluorescence units) of Amplex Red. A Two-Way ANOVA model did not find significant interaction between treatment and concentration level. The difference between treatments (*wzy* or wild type LPS) was also not significant at any concentration. The multiple comparisons show that ROS production is not significant among 5 µg, 10µg and 12.5µg LPS doses. The only significant difference was between 10µg and 20µg. Graphs represent the mean of 5 biological replicates per treatment ± standard error of the mean. Treatments with the same

letters over the bars belong to the same statistical group ($P < 0.05$, two-way ANOVA, n=5/treatment).

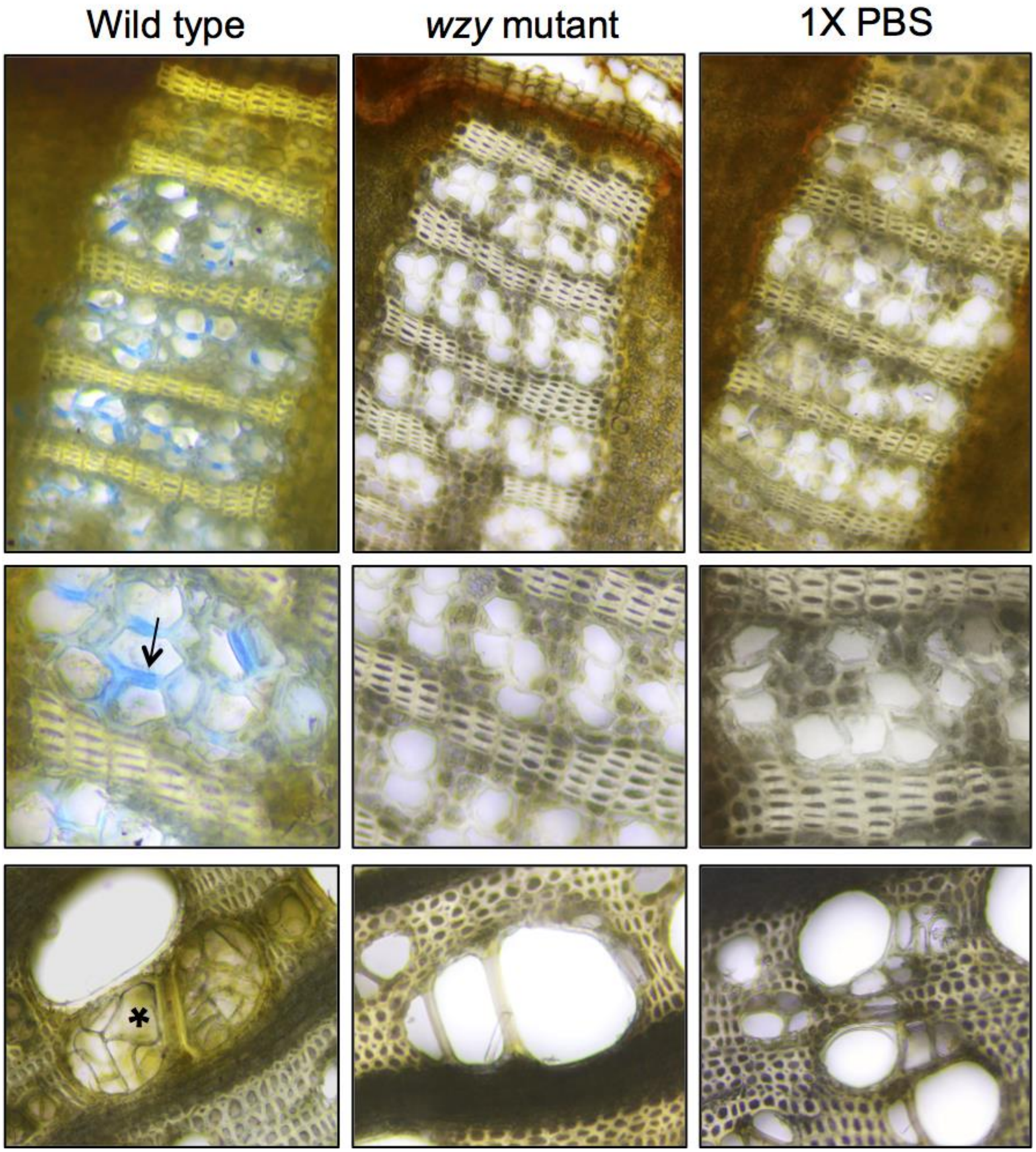

Supplementary Figure 2. Callose and suberin deposition are more prevalent in wild type-infected vines.

Images represent grapevines at 18 weeks post-inoculation, inoculated with wild type *Xf* cells,

wzy cells, or 1X PBS buffer. Wild type-inoculated plants exhibited widespread callose deposition in the phloem tissue (appears as blue color, indicated by arrow). In addition, there was pronounced deposition of suberin in xylem vessels (indicated by gold color), especially in vessels with multiple tyloses (*). No callose or suberin was present in the stems of *wzy* or 1X PBS-inoculated vines.

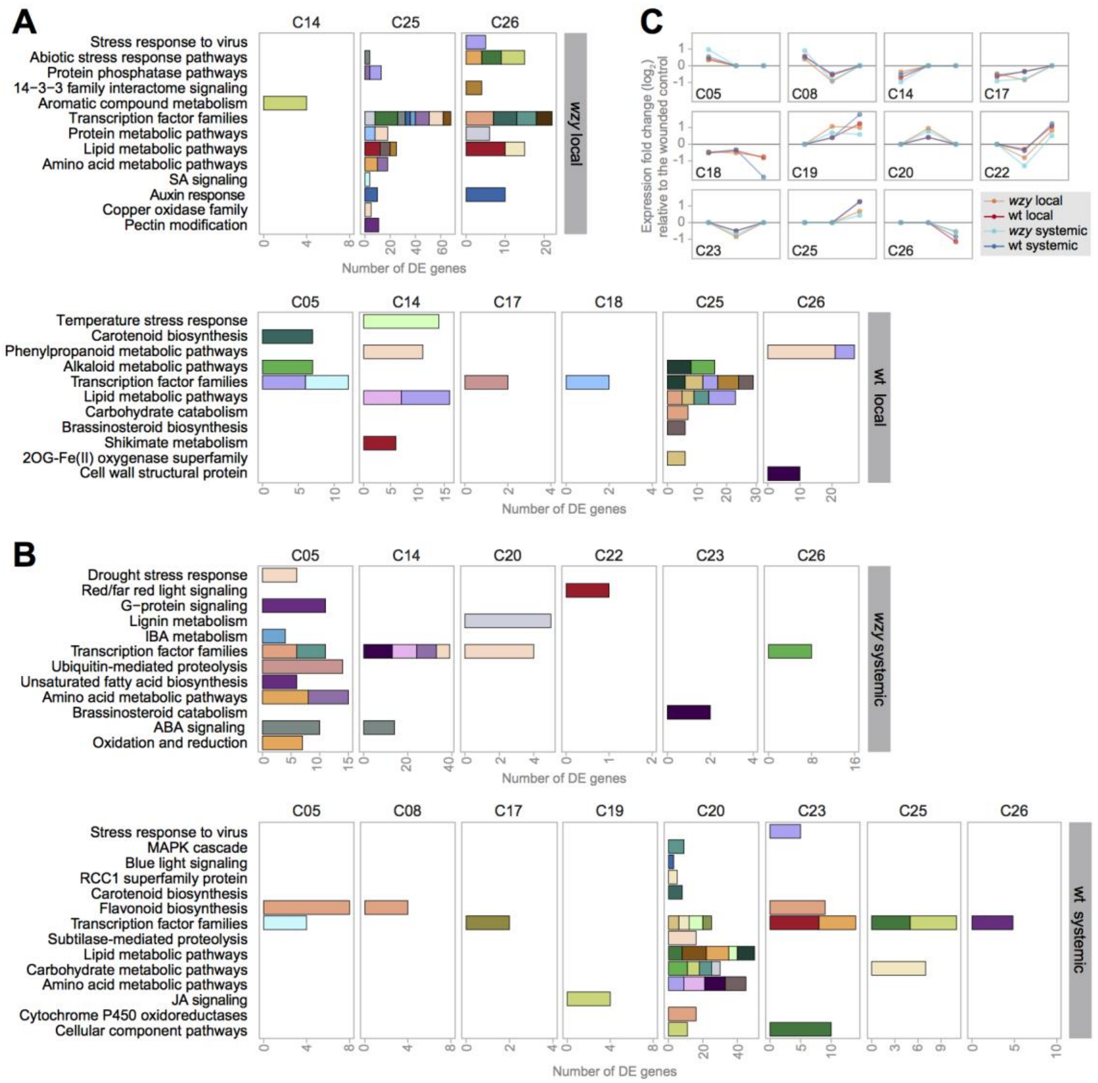

Supplementary Figure 3. Temporal dynamics of transcriptional responses to *Xf* wild type or *wzy* mutant strains in local and systemic tissue. Enriched grape functional pathways ($P < 0.05$, hypergeometric test, n=3/treatment) in differentially expressed (DE, $P < 0.05$, DESeq differential expression test, n=3/treatment) gene clusters representing local **(A)** or systemic **(B)** responses to *Xf* inoculation. Only enriched pathways related to grapevine immune responses and that were unique to wild type (wt) or *wzy* inoculations are depicted. Colored stacked bars

represent individual pathways. **(C)** Patterns of expression of gene clusters enriched in functional pathways with biological relevance. Lines represent the medoids for each cluster. Dots represent expression fold changes of each medoid (log2) at a given time point post-inoculation (in order: 48 h, 1 week, and 4 weeks) when compared to 1X PBS controls. The complete dataset, including the color legend for each pathway, is available in Supplementary Data 4.

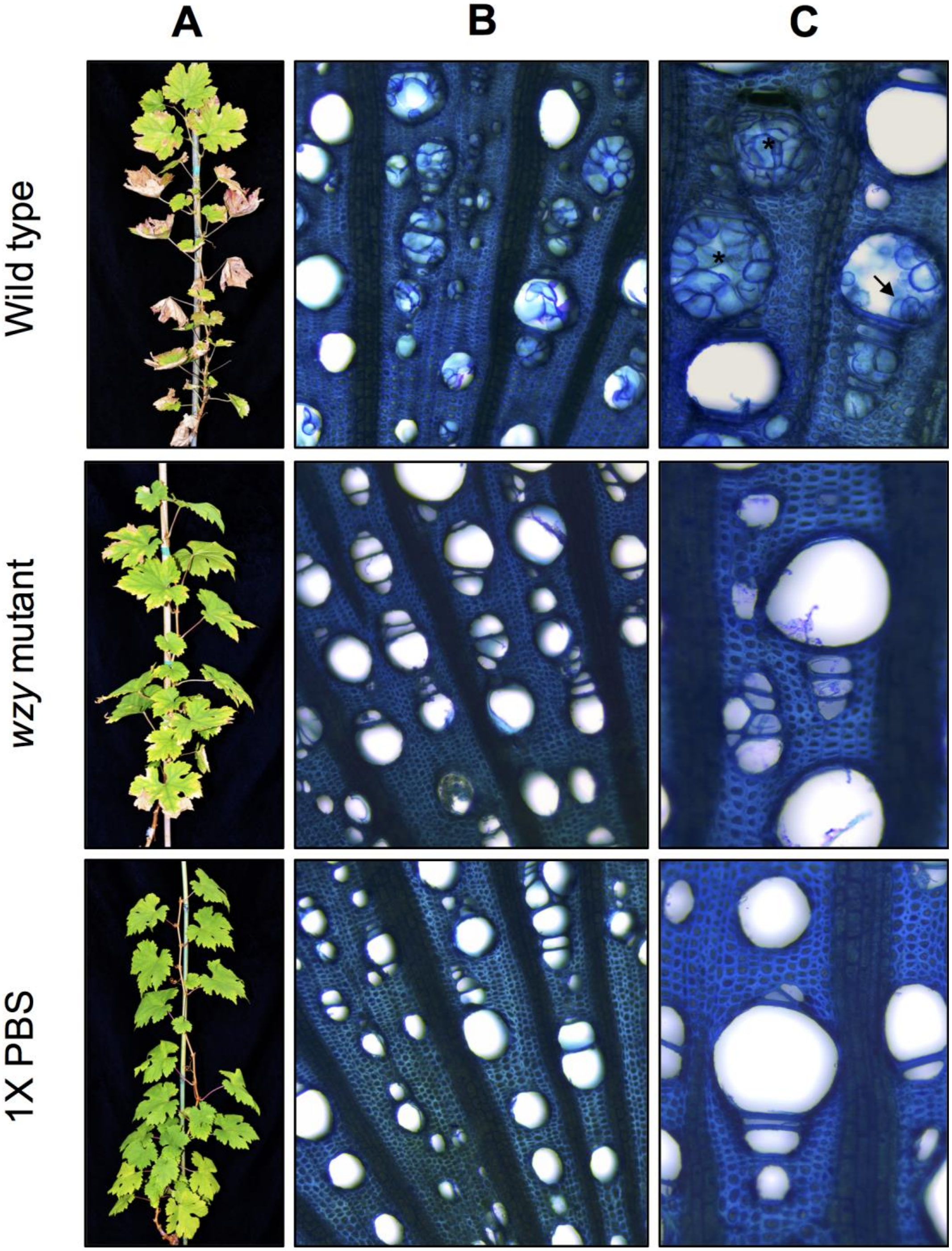
A
B
C
Wild type
wzy mutant
1X PBS

Supplementary Figure 4. Tylose development in PD-infected grapevines. Images represent grapevines at 18 weeks post-inoculation, inoculated with wild type *Xf* cells, *wzy* cells, or 1X PBS buffer. **(A)** Representative images of PD progress prior to histological examination. **(B)** Micrograph showing tylose production in cross sections of grapevine xylem (brightfield stained with Toluidine Blue O). **(C)** Close-up of xylem vessels showing complete occlusion with multiple tyloses (*) in wild type-inoculated vines. A few small tyloses also occurred in these vines (closed arrowheads). Tyloses were largely absent in the xylem vessels of *wzy*-inoculated vines. No tyloses were present in the stems of 1X PBS-inoculated vines.

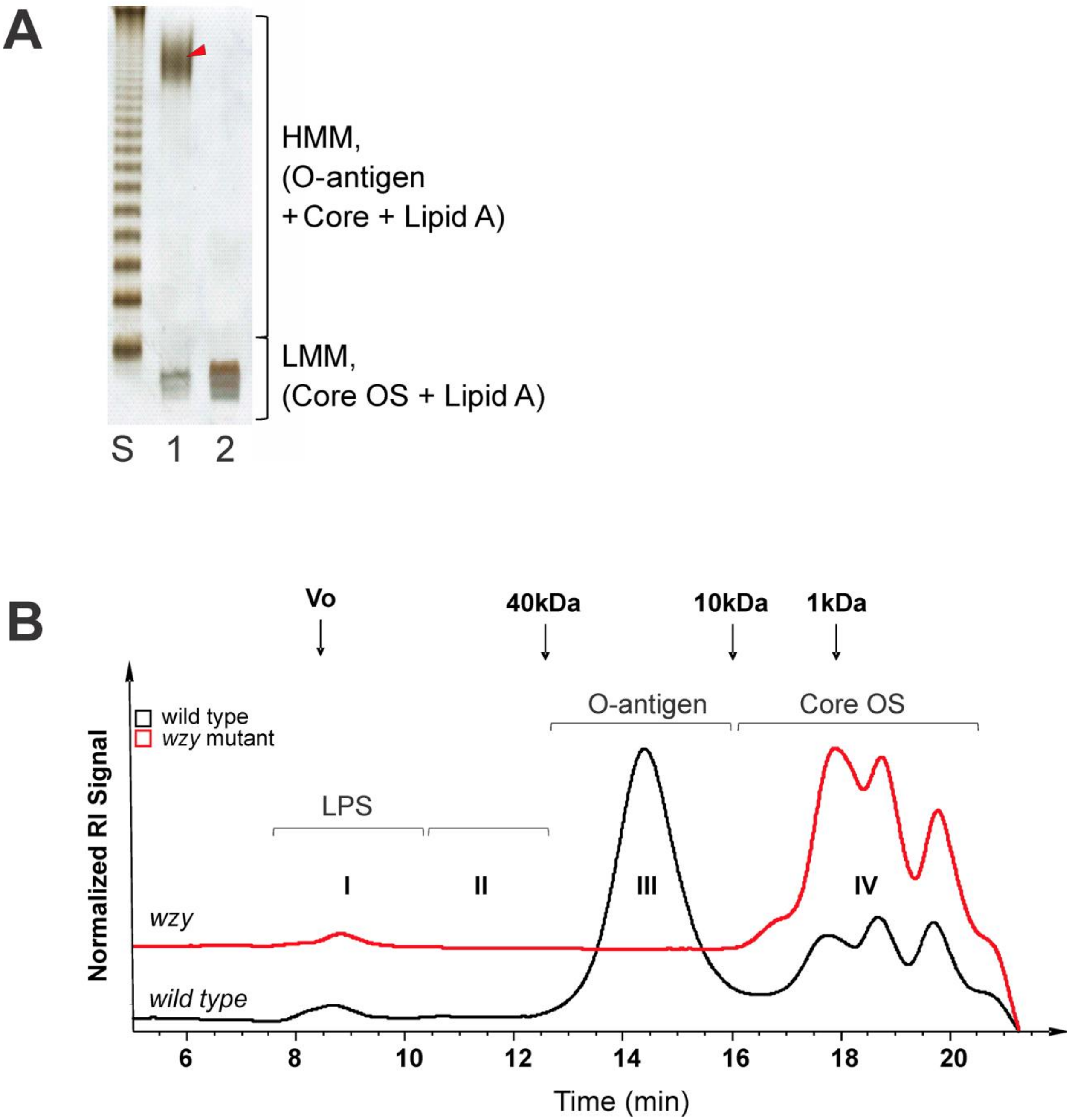

Supplementary Figure 5. Characterization of *Xylella fastidiosa* LPS. (A) DOC-PAGE analysis of LPS isolated from *Xf* wild type and *wzy* cells. Lane S = *Salmonella enterica* s.

Typhimurium, smooth LPS; Lane 1 = Wild type; Lane 2 = *wzy* mutant. Red arrow indicates the presence of high molecular mass (HMM) O-antigen that is not observed in the *wzy* LPS. **(B)** SEC chromatograms of polysaccharides liberated from LPS of *Xf* wild type (black) and *wzy* (red). Standard dextrans of 40,000, 10,000 and 1,000 Da were used for calibration of the Superose 12.

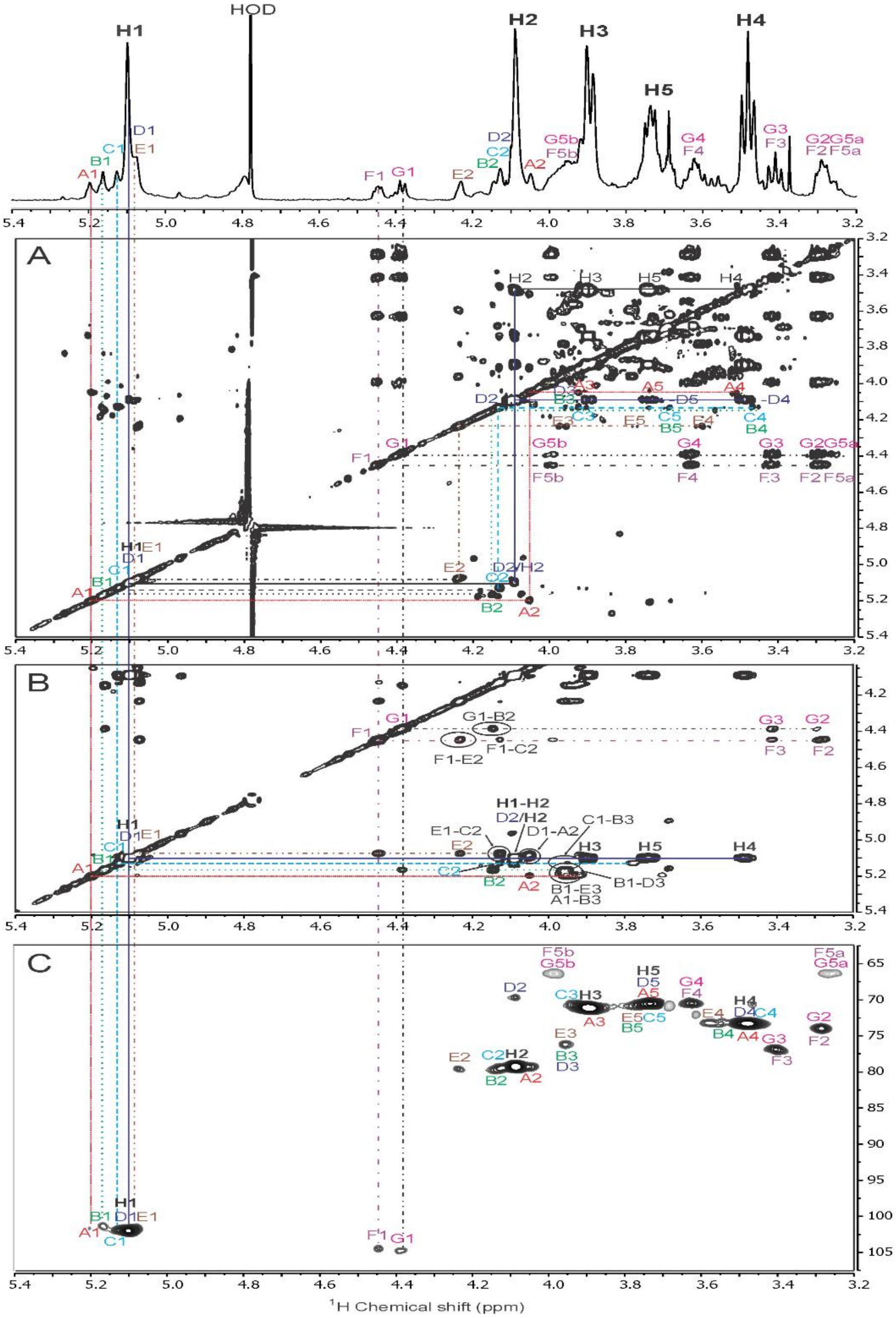

Supplemental Figure 6. 1D and 2D nuclear magnetic resonance spectroscopy. The 1D ^{1}H

NMR and 2D TOCSY-150 ms (A), NOESY-200 ms (B) and HSQC spectra (C) of O-antigen isolated from *X. fastidiosa*, recorded in D_2O at 25°C. The CH_3 signals were observed at ~ δ 1.30, but were not included in the figure. Cross-peaks belonging to the same scalar-coupling network are indicated near dotted lines starting from the corresponding diagonal peaks.

SUPPLEMENTARY TABLES

Supplementary Table 1. Glycosyl components identified in the LPS of *X. fastidiosa* wild type and *wzy* mutant.

	Glycosyl residues (mol %)							
**LPS**	**Rha**	**Xyl**	**Man**	**Glc**	**GalA**	**GlcN**	**Kdo***	**2-Me-Rha**
**Wild type**	**82**	**12**	**2**	**2**	**2**	**0.3**	**+**	**<0.1**
wzy	**19**	**2**	**4**	**15**	**53**	**8**	**+**	**nd**

Legend: * Identified in TMS analysis but not quantified; 2-Me-Rha was identified in trace amounts (below 0.1%) only in the parent strain.

Supplementary Table 2. ^{1}H and ^{13}C NMR chemical shifts of the O-polysaccharides, recorded in D_2O, at 25°C.

	**Residue**	**^{1}H / ^{13}C chemical shifts (δ)**					
		**1**	**2**	**3**	**4**	**5**	**6**
A	→2)-α-L-Rha*p*-(1→	5.20	4.05	3.92	3.51	3.74	1.31
		102.0	79.3	71.0	73.3	70.5	18.0
B	→2,3)-α-L-Rha*p*-(1→	5.16	4.15	3.95	3.55	3.78	1.29
		101.2	79.6	76.2	73.2	70.5	18.0

C	→2)-α-L-Rha*p*-(1→	5.13	4.13	3.92	3.46	3.71	1.28
		102.0	79.6	70.7	70.7	70.5	18.0
D	→3)-α-L-Rha*p*-(1→	5.10	4.08	3.95	3.48	3.74	1.30
		102.0	69.7	76.2	73.3	70.5	18.0
E	→2,3)-α-L-Rha*p*-(1→	5.08	4.23	3.96	3.58	3.78	1.29
		102.0	79.6	76.2	73.2	70.8	18.0
F	β-D-Xyl*p*-(1→	4.45	3.28	3.40	3.62	3.98/3.26	-
		104.5	74.0	76.9	70.4	66.3	
G	β-D-Xyl*p*-(1→	4.38	3.28	3.40	3.62	3.98/3.26	-
		104.7	74.0	76.9	70.4	66.3	
H	→2)-α-L-Rha*p*-(1→	5.10	4.09	3.90	3.48	3.74	1.28
		103.2	79.3	71.1	73.3	70.5	18.0

SUPPLEMENTARY REFERENCES

1. Senthil-Kumar M & Mysore KS (2013) Nonhost resistance against bacterial pathogens: retrospectives and prospects. *Annual review of phytopathology* 51:407-427.

2. Sun Q, Sun Y, Walker MA, & Labavitch JM (2013) Vascular occlusions in grapevines with Pierce's disease make disease symptom development worse. *Plant physiology* 161(3):1529-1541.

3. Senchenkova SN, Shashkov AS, Laux P, Knirel YA, & Rudolph K (1999) The O-chain polysaccharide of the lipopolysaccharide of *Xanthomonas campestris* pv. begoniae GSPB 525 is

a partially L-xylosylated L-rhamnan. *Carbohydr Res* 319(1-4):148-153.

4. Ciucanu I & Kerek F (1984) A simple and rapid method for the permethylation of carbohydrates. *Carbohydrate Research* 131:209-217.

5. Gerwig GJ, Kamerling JP, & Vliegenthart JFG (1978) *Carbohydrate Research* 62:349-357.
