# Supplementary material for: Lipopolysaccharide O-antigen delays plant innate immune recognition of Xylella fastidiosa
Source: Nat Commun. 2018 Jan 26;9:390. doi: 10.1038/s41467-018-02861-5 (PMC5786101; doi:10.1038/s41467-018-02861-5)
Supplement: Supplementary file 2 — Description of Additional Supplementary Files [file 41467_2018_2861_MOESM2_ESM.pdf]

Description of Supplementary Files

File Name: Supplementary Data 1
Description: **Summary of parsed and mapped reads obtained in the two RNAseq studies.**

File Name: Supplementary Data 2
Description: **Validation of the expression of a set of genes from the RNAseq study on early grapevine responses using qRT-PCR.** qRT-PCR validation of a subset of genes from the RNAseq study on early grapevine responses to wild type and *wzy* mutant *Xf*. The results are shown as fold changes (log2), and the corresponding *P* values are provided.

File Name: Supplementary Data 3
Description: **Enriched grape functional pathways (P < 0.05) among genes upregulated during *wzy* and/or wild type early infection (8h and 24hr) post-inoculation.**

File Name: Supplementary Data 4
Description: **Enriched grape functional pathways (P < 0.05) among genes upregulated during *Xf* wild type or *wzy* mutant infections in local and systemic tissue over time.**

File Name: Supplementary Data 5
Description: **Primer sequences of grapevine genes of biological importance during *X. fastidiosa* infections.**
